# Longitudinal method comparison: modeling polygenic risk for post-traumatic stress disorder over time in individuals of African and European ancestry

**DOI:** 10.3389/fgene.2024.1203577

**Published:** 2024-05-16

**Authors:** Kristin Passero, Jennie G. Noll, Shefali Setia Verma, Claire Selin, Molly A. Hall

**Affiliations:** ^1^ Virginia Institute of Psychiatric and Behavioral Genetics, Virginia Commonwealth University, Richmond, VA, United States; ^2^ Department of Psychology, Mount Hope Family Center, University of Rochester, Rochester, NY, United States; ^3^ Department of Pathology and Laboratory Medicine, University of Pennsylvania, Philadelphia, PA, United States; ^4^ Center for Childhood Deafness, Language, and Learning, Boys Town National Research Hospital, Omaha, NE, United States; ^5^ Department of Genetics and Institute for Biomedical Informatics, University of Pennsylvania, Philadelphia, PA, United States

**Keywords:** longitudinal analysis methods, repeated measures, simulation study, polygenic risk scores, post-traumatic stress disorder, longitudinal method comparison

## Abstract

Cross-sectional data allow the investigation of how genetics influence health at a single time point, but to understand how the genome impacts phenotype development, one must use repeated measures data. Ignoring the dependency inherent in repeated measures can exacerbate false positives and requires the utilization of methods other than general or generalized linear models. Many methods can accommodate longitudinal data, including the commonly used linear mixed model and generalized estimating equation, as well as the less popular fixed-effects model, cluster-robust standard error adjustment, and aggregate regression. We simulated longitudinal data and applied these five methods alongside naïve linear regression, which ignored the dependency and served as a baseline, to compare their power, false positive rate, estimation accuracy, and precision. The results showed that the naïve linear regression and fixed-effects models incurred high false positive rates when analyzing a predictor that is fixed over time, making them unviable for studying time-invariant genetic effects. The linear mixed models maintained low false positive rates and unbiased estimation. The generalized estimating equation was similar to the former in terms of power and estimation, but it had increased false positives when the sample size was low, as did cluster-robust standard error adjustment. Aggregate regression produced biased estimates when predictor effects varied over time. To show how the method choice affects downstream results, we performed longitudinal analyses in an adolescent cohort of African and European ancestry. We examined how developing post-traumatic stress symptoms were predicted by polygenic risk, traumatic events, exposure to sexual abuse, and income using four approaches—linear mixed models, generalized estimating equations, cluster-robust standard error adjustment, and aggregate regression. While the directions of effect were generally consistent, coefficient magnitudes and statistical significance differed across methods. Our in-depth comparison of longitudinal methods showed that linear mixed models and generalized estimating equations were applicable in most scenarios requiring longitudinal modeling, but no approach produced identical results even if fit to the same data. Since result discrepancies can result from methodological choices, it is crucial that researchers determine their model *a priori*, refrain from testing multiple approaches to obtain favorable results, and utilize as similar as possible methods when seeking to replicate results.

## 1 Introduction

Cross-sectional studies have been the driving force behind developments in genome research, having given rise to the genome-wide association study (GWAS) and analogous methods, such as phenome-wide or environment-wide association studies (Hall et al., 2016). However, they have limited capability to evaluate genetic influences on the development of complex diseases as cross-sectional data lack the information necessary to model change (Singer and Willett, 2003). However, data with repeated phenotype measures over time not only allow the assessment of how the genome affects the phenotype at a given time point but can also describe how the trait progresses over time and how genetic risk alters this trajectory (Singer and Willett, 2003). Repeated measures, or longitudinal data, are clustered data, wherein a “cluster” is an individual with repeated measurements. It is expected that repeated measures within a sample will be correlated (dependent) (Gibbons et al., 2010), which violates the independence assumption of commonly used linear or logistic regression models. Ignoring this violation by analyzing clustered data without accommodating dependency could inflate the false positive rate (FPR). For instance, Musca et al. (2011) found that applying a *t*-test to dependent data produced an FPR of above 50% even if the dependency was low to moderate (Musca et al., 2011).

Many methods for analyzing longitudinal data exist, but the currently favored approaches are linear mixed models (LMMs) and generalized estimating equations (GEEs) (Gibbons et al., 2010; Garcia and Marder, 2017; Woodard, 2017). LMMs represent dependency by modeling “fixed” and “random” effects (i.e., the intercept or slope parameters defined in a regression equation) (McNeish et al., 2017). “Fixed” effects are shared by all clusters and can be thought as population-level effects (McNeish et al., 2017). A “random” effect is cluster-specific and allows each cluster to deviate uniquely from the fixed effects (McNeish et al., 2017), thereby accommodating the similarity from correlated repeated measures. LMMs are advantageous because they model time-invariant and time-variant predictors, treat time as a continuous variable, can represent two or more levels of clustering, and have less strict missingness assumptions (Gibbons et al., 2010; Garcia and Marder, 2017; Woodard, 2017). Nevertheless, they have many assumptions regarding the distribution of predictors, random effects, and residuals (McNeish et al., 2017), violations of which could negatively affect analytical performance (Dieleman and Templin, 2014). Generalized LMM extensions to analyze noncontinuous outcomes are also computationally expensive (Garcia and Marder, 2017; McNeish et al., 2017). Furthermore, they estimate additional parameters—the variance–covariance of random effects—that usually are not of interest to the researcher (McNeish et al., 2017). GEEs also model time-varying predictors and continuous time but have fewer assumptions than LMMs (Gibbons et al., 2010; Garcia and Marder, 2017; McNeish et al., 2017; Woodard, 2017). Instead, GEEs compute population-average coefficient estimates while separately estimating group dependency via a working correlation matrix that is used to correct parameter estimates (Gibbons et al., 2010; Garcia and Marder, 2017; McNeish et al., 2017; Woodard, 2017). Some limitations of GEEs are their decreased tolerance of missing data and use of quasi-likelihood, rather than maximum likelihood, estimation (Gibbons et al., 2010; Garcia and Marder, 2017; McNeish et al., 2017; Woodard, 2017). Popular likelihood-based measures or tests—the likelihood ratio test (LRT), Akaike information criterion, etc.—cannot be applied to GEEs because they are quasi-likelihood methods. Furthermore, open-access implementations of GEEs in R and Python also do not specify more than two levels of clustering (Halekoh et al., 2006; Seabold and Perktold, 2010; Carey et al., 2022).

Other approaches applicable to longitudinal data analysis are cluster-robust standard errors (CRSEs), fixed-effects (FE) models, and aggregate regression (AGG). CRSEs adjust coefficient standard errors to reflect dependency and make fewer assumptions than LMMs and GEEs (McNeish et al., 2017; Bauer et al., 2020). A linear or logistic regression model is fit to the dependent data, and then standard errors are re-calculated using the CRSE approach, which incorporates the dependency within clusters into the standard error estimation. CRSEs accommodate two levels of clustering and, as they only adjust the standard errors, they require that the regression model is correctly specified to ensure unbiased coefficient estimates (McNeish et al., 2017). The FE model accommodates group dependency by adding cluster membership to the regression model as a dummy-encoded covariate (Bauer et al., 2020). In FE models, inference cannot be done at the group level since all between-group differences are adjusted out of the model after including cluster membership as a covariate. However, FE models are conceptually simple, easy to implement, and outperform LMMs in situations with few groups (Dieleman and Templin, 2014; McNeish and Stapleton, 2016). AGG consolidates repeated measurements on an individual into a single value by averaging them over time (Aarts et al., 2014). This reduces clusters to single independent data points, and the new dataset can be analyzed with traditional methods that assume independence. However, the AGG approach necessarily precludes investigation into how a trait develops over time.

Previous GWASs have analyzed longitudinal traits with LMMs (Smith et al., 2010; Wendel et al., 2021) or GEEs (Honne et al., 2016). However, many GWASs opted to simplify the repeatedly measured phenotype into a single measure for analysis with methods assuming independence (Cousminer et al., 2013; Hoffmann et al., 2018; Alves et al., 2019; Tan et al., 2021). Longitudinal studies incorporating polygenic risk scores (PRSs) have been performed, using a wide variety of techniques. The most prevalent approaches are the LMM (Liu et al., 2021; Choe et al., 2022; Machlitt-Northen et al., 2022; Segura et al., 2022; Tomassen et al., 2022), GEE (Ihle et al., 2020; Tsapanou et al., 2021; Tomassen et al., 2022), and, for dichotomous outcomes, time-to-event analysis (Paul et al., 2018; Khera et al., 2019; Ihle et al., 2020; Liu et al., 2021; Ajnakina et al., 2022). Time-to-event analysis is used to investigate whether and when a change in phenotype status occurs, such as a switch from control to case status (Singer and Willett, 2003). However, time-to-event data have unique characteristics that require analysis by methods other than LMMs, GEEs, etc., and, as such, are not the focus of this study [for an overview, see Le-Rademacher and Wang, 2021; Schober and Vetter, 2018; Le-Rademacher and Wang, 2021; Schober and Vetter, 2018)]. Aggregate regression has also been used in longitudinal PRS studies, as by Waszczuk et al. (2022), who found various significant associations between various mental health PRSs and average post-traumatic stress disorder (PTSD) symptoms over time (Waszczuk et al., 2022). However, they complimented this approach with a latent trajectory analysis to show whether the PRSs also predicted the PTSD trajectory class (Waszczuk et al., 2022).

In this study, we investigated various longitudinal modeling approaches to determine how they compare when analyzing trajectory changes of a continuous, repeatedly measured phenotype. We evaluated the power, FPR, and estimation accuracy/precision of LMMs, GEEs, CRSEs, AGG, and FE models alongside naïve linear regression (NLR) with a simulation study. For this simulation study, not all methods explicitly modeled trait development over time. The AGG and NLR approaches always ignored changes in the dependent variable but were included to emphasize the differing results one may observe when discounting changes over time. NLR did not include any adjustments to account for modeling time or dependency and, as such, served as the baseline to which all other methods could be compared.

Using the simulation results, we applied the most accurate methods to a longitudinal cohort of African and European ancestry to examine the genetic and environmental influences on PTSD symptoms over time. The results showed that the analytical strategy and model design greatly influence results and interpretation. In our simulation, NLR and FE approaches had inflated false positive rates when analyzing a predictor that was fixed over time (e.g., genetics), whereas the viability of all other methods depended on the characteristics of the dataset being studied. In our longitudinal cohort, African-ancestry and European-ancestry participants showed different associations with PTSD symptoms over time. Researchers interested in genetic longitudinal studies need to consider the trade-offs between power, false positives, and estimation and accommodate potential time-varying effects in their analysis to procure accurate, reliable results.

## 2 Materials and methods

### 2.1 Simulation study comparing longitudinal data analysis methods

To compare the performance of longitudinal data analysis (LDA) methods, we designed a simulation study which generated longitudinal data and applied six methods in statistical hypothesis tests. The simulated longitudinal data consisted of repeated measures on individuals and disregarded higher levels of clustering. We simulated fixed effects and random effects in each longitudinal dataset to produce group dependency (fixed and random effects describe effects shared between groups and group-specific effects, respectively). The methods studied were NLR, CRSE, AGG, FE, LMM, and GEE.

To implement the simulations, we wrote R functions to generate data and apply the chosen analytical methods. These functions are stored in a custom package, *LDA simulations*, available on GitHub (https://github.com/HallLab/ldasimulations). We used base R functions to randomly generate variables. The fitting of models used various statistical R packages. The *stats* general linear model function was used to implement NLR and AGG models. For the CRSE approach, we first fit linear regression, and then the CRSE adjustment was applied to the output. CRSE calculations were provided by *lmtest* (Zeileis and Hothorn, 2002) and the cluster-robust variance estimator from the *sandwich* package (Zeileis, 2004; Zeileis, 2006; Zeileis et al., 2020); CRSEs utilized the default degrees of freedom and applied an HC1 sample-size correction. LMMs were implemented using *lme4* (Bates et al., 2015) alongside *lmerTest* for the calculation of *p*-values (Kuznetsova et al., 2017). GEEs were implemented using *geepack* (Halekoh et al., 2006). All simulations were run in R version 4.1.

#### 2.1.1 Simulation of longitudinal datasets and application of LDA methods to the simulated data

Phenotype trajectories can be described by their initial value and their change over time. The rate of change of the phenotype is necessarily a function of time but can also be altered by other variables, which is often of interest to the researcher (e.g., does greater polygenic risk increase the rate of change of body weight?). To examine situations where phenotype change over time is affected by a predictor X , we simulated longitudinal data consisting of a response Y , whose trajectory was determined by the effects of X , Time , and a time-varying effect X×Time . The trajectory of Y was also affected by a cluster-specific random intercept (RI) and residual error. We varied multiple parameters of the simulated longitudinal data to compare method performance across different sample sizes, effect sizes, strengths of dependency, predictor types, and response linearity (examples of simulated data are given in Supplementary Figure S1). The datasets had *i* individuals measured at *t* = 4 time points, where *i* ranged from 50 to 500 (Table 1A). The predictor X could be fixed over time (“time-invariant”) or vary across time (“time-variant;” Table 1A). If time-invariant, then the value of X was the same for all observations within a cluster. Alternatively, for a time-variant predictor, the values of X were independently generated for individual *i* at time *t* and could vary within the cluster. To reflect different variable types found in natural data, X was either drawn from (1) a binomial distribution with a 50% probability of “exposure” or (2) a standard normal distribution (Table 1A). The final simulated predictor was the interaction X×Time . This term indicates that the effect of X varies over time, affecting the rate of change of the response trajectory. The true effects between X , Time , and X×Time and the response Y were described by $\beta_{1}$ , $\beta_{2}$ , and $\beta_{3}$ , respectively. These coefficients were all equal and were set to {0, 0.05, 0.1, and 0.3} (Table 1A). The datasets where β=0 were null datasets used to assess false positive rates. To simulate dependency within the data, a RI was generated for each cluster. This produced similarity between the observations on an individual. The RI was drawn from a normal distribution centered at zero with a variance of $\sigma_{g}^{2}$ , the between-group variance. A residual error (*e*) was generated uniquely for every observation in the simulated dataset. The residuals came from a normal distribution with a mean of zero and variance $\sigma_{e}^{2}$ . The strength of dependency within the dataset was determined by the ratio of $\sigma_{g}^{2}$ to $\sigma_{e}^{2}$ . Higher $\sigma_{g}^{2}$ relative to $\sigma_{e}^{2}$ indicates greater dependency as the total variance comprises more between-cluster variation. The proportion of variation due to between-group differences can be used to calculate a measure of dependency, the intraclass correlation coefficient (ICC), with the formula $ICC=\sigma_{g}^{2}/\left(\sigma_{g}^{2}+\sigma_{e}^{2}\right)$ . The ICC ranges from 0 to 1, where ICC = 0 means data are independent, while ICC = 1 means all values are identical within a cluster. We arbitrarily set the sum of $\sigma_{g}^{2}$ and $\sigma_{e}^{2}$ to 10 and then generated the RI and *e* terms to meet ICCs of 0.1, 0.5, and 0.9 (Table 1A). The values of X , Time , X×Time , RI, and *e* were first generated for each individual *i* at time *t*, and then the values were summed with their appropriate effect sizes to produce the response Y . As all methods under consideration assume a linear relationship between the predictors and response, we simulated a relationship that was linear (Eq. 1, exponential Eq. 2, or parabolic Eq. 3; Table 1A). The latter cases (Eqs 2–3) produce data with a nonlinear relationship to investigate how methods compare when all are disadvantaged by assumption violations. The nonlinearity of the predictor–response relationship in Eqs 2–3 was likely to induce estimate bias by the applied methods. All response-generating formulas (Eqs 1–3) had an intercept term $\beta_{0}$ , which was set to 1.

$$Y_{it}=\beta_{0}+\beta_{1}X_{it}+\beta_{2}{Time}_{it}+\beta_{3}X_{it}{Time}_{it}+{RI}_{i}+e_{it},$$ (1)

$$Y_{it}=\beta_{0}+e^{\beta_{1}X_{it}+\beta_{2}{Time}_{it}+\beta_{3}X_{it}{Time}_{it}}+{RI}_{i}+e_{it},$$ (2)

$$Y_{it}=\beta_{0}+\beta_{1}X_{it}+\beta_{2}{{Time}_{it}}^{2}+\beta_{3}{\left(X_{it}{Time}_{it}\right)}^{2}+{RI}_{i}+e_{it}.$$ (3)

**TABLE 1:** Simulation parameters and values for (A) primary simulation, (B) limited simulation without time-varying effects, and (C) limited simulation with a correctly specified cluster-robust standard error (CRSE) model.

Parameters	Values		
	(A) Time-varying effects	(B) No time-varying effects	(C) Time-varying effects + correctly specified CRSE
Intraclass correlation coefficient (ICC)	0.1, 0.5, and 0.9	0.5	0.1, 0.5, and 0.9
$\sigma_{g}^{2}:\sigma_{e}^{2}$	1:9, 5:5, and 9:1	5:5	1:9, 5:5, and 9:1
# Clusters (*i*)	50, 75, 100, 200, and 500	50 and 100	50 and 100
# Time points (*t*)	4	4	4
Time increment	1 unit	1 unit	1 unit
Intercept $\left(\beta_{0}\right)$	1	1	1
Fixed effects $\left(\beta_{1},\beta_{2},\beta_{3}\right)$	0, 0.05, 0.1, and 0.3	0 and 0.3	0, 0.1, and 0.3
Response linearity	Linear, exponential, and parabolic	Linear and exponential	Linear and exponential
Predictor ( X ) type	*Bin*(*n*, 0.5), *N* (0,1)	*N* (0,1)	*N* (0,1)
	Time-invariant and time-variant	Time-invariant and time-variant	Time-invariant and time-variant
Response-generating variables	X,Time,X×Time	X,Time	X,Time,X×Time

In total, there were 720 possible combinations of parameters (Table 1A); each was generated 1,000 times. After a dataset was generated, the six methods—NLR, CRSE, AGG, FE, LMM, and GEE—were applied. The resulting coefficient estimates and *p*-values were extracted for comparison. NLR was fit with the model $Y=\beta_{0}+\beta_{1}X$ , and CRSEs were also applied a linear regression fit with $Y=\beta_{0}+\beta_{1}X$ . Before the AGG model was applied, the terms Y and X were averaged by group; then, the model $\bar{Y}=\beta_{0}+\beta_{1}\bar{X}$ was fit with the newly summarized data. The FE model was fit as $Y=\beta_{0}+\beta_{1}X+\beta_{2}Time+\beta_{3}X\times Time+\beta_{4}{Group}_{2}\ldots +\beta_{i+4}{Group}_{i}$ . Both the LMM and GEE were fit with the model $Y=\beta_{0}+\beta_{1}X+\beta_{2}Time+\beta_{3}X\times Time$ . The LMM assumed a random intercept per cluster. The working correlation structure assumed by the GEE was “exchangeable,” in which each pair of observations within a cluster is equally correlated. The implemented NLR, AGG, and CRSE models were underparameterized regarding the true data-generating model (Eqs 1–3) as they did not model Time or X×Time explicitly. Thus, they could not assess phenotype change over time, but we evaluated such models to observe whether ignoring real effects of Time or X×Time caused a performance deficit. All characteristics of these simulated datasets are given in Table 1A.

If the data contain true effects of X , Time , and their interaction X×Time , this confers an analytical advantage to methods explicitly modeling all three terms as ignoring their true effects could bias estimates of the effect of X . However, some predictor X may affect the average response at each time point but not the change in the phenotype trajectory (i.e., X does not have a time-varying effect X×Time ). In such a case, methods that ignore Time (e.g., AGG method) may be appliable if no X×Time effect exists. Therefore, we simulated data wherein the response variable was generated without the X×Time interaction to represent data without time-varying effects of X . We then compared methods that either did or did not explicitly model Time and X×Time . In this simulation without time-varying effects, we also varied the parameters of sample size, effect size, predictor type, and response linearity. The trajectory of Y was determined by X , Time , a cluster-specific RI, and residual error. Datasets had *i* individuals measured at *t* = 4 time points, where *i* was either 50 or 100 (Table 1B). The predictor X came from a standard normal distribution and could be time-invariant (fixed across time) or time-variant (changing across time) (Table 1B). $\beta_{1}$ and $\beta_{2}$ were the effects of X and Time , respectively. For all simulations, $\beta_{1}=\beta_{2}$ and were 0 or 0.3, with null values included to test the FPR (Table 1B). The RI and residual error *e* were drawn from normal distributions centered at zero with variances of $\sigma_{g}^{2}$ and $\sigma_{e}^{2}$ , respectively. The values of $\sigma_{g}^{2}$ and $\sigma_{e}^{2}$ summed to 10 and were such that the ICC equaled 0.5 (Table 1B). The X , Time , RI, and *e* values were generated first and then summed with their respective effect sizes to produce the response Y . The relationship between Time and the response was either linear (Eq. 4) or exponential (Eq. 5). Only Time was exponentiated to observe whether bias in the estimation of X was apparent in methods that did not explicitly model Time . The intercept term, $\beta_{0}$ , was set to 1 in all response-generating functions (Eqs 4–5).

$$Y_{it}=\beta_{0}+\beta_{1}X_{it}+\beta_{2}{Time}_{it}+{RI}_{i}+e_{it},$$ (4)

$$Y_{it}=\beta_{0}+\beta_{1}X_{it}+e^{\beta_{2}{Time}_{it}}+{RI}_{i}+e_{it}.$$ (5)

Data corresponding to each of these 16 possible parameter sets (Table 1B) were replicated 1,000 times. We applied NLR, AGG, CRSE, and two LMMs to these simulated datasets. The former three methods modeled X but not Time . NLR and CRSE were fit with the model $Y=\beta_{0}+\beta_{1}X$ . For the AGG model, Y and X were averaged by group, and then the model $\bar{Y}=\beta_{0}+\beta_{1}\bar{X}$ was applied. The first LMM was fit with $Y=\beta_{0}+\beta_{1}X+\beta_{2}Time$ , which directly matches the response-generating Eq. 4. The second LMM was overparameterized and fit with $Y=\beta_{0}+\beta_{1}X+\beta_{2}Time+\beta_{3}X\times Time$ . Both LMMs assumed a random intercept. To differentiate between the LMMs in simulations without time-varying effects, we refer to the correctly specified model as the “LMM,” while the overparameterized model is the “LMM + interaction” (LMM+Int) model in our figures and tables. The NLR, AGG, CRSE, and LMM+Int approaches were all designed to be compared to the LMM approach, which was identical to the linear data-generating formula in Eq. 4 and, therefore, expected to have the best performance. Coefficient estimates and their *p*-values were extracted for all available terms in the model.

In the aforementioned simulations, the CRSE was implemented upon a regression that did not model Time or the X×Time interaction and, thus, could not analyze the phenotype trajectory change. However, the CRSE is flexible in that it can be applied to any model formulation, provided that group membership is known and only two levels of clustering exist (e.g., repeated measures on independent individuals). We wanted to assess the performance of the CRSE when applied to a model that correctly fit X , Time , and X×Time . Longitudinal data were simulated with full time-varying effects (Eqs 1–2), and then CRSE performance was tested when applied to a regression with all response-generating terms. The datasets contained *i* individuals, where *i* = 50 or 100, measured at *t =* 4 time points (Table 1C). We generated a time-invariant or time-variant standard normal predictor X (Table 1C). The effects of X , Time , and X×Time , represented by $\beta_{1}$ , $\beta_{2}$ , and $\beta_{3}$ , respectively, were equal and set to {0, 0.1, and 0.3} (Table 1C). Datasets with null effects ( β=0 ) were generated to evaluate the FPR. The response Y also depended on an RI and residual error *e*. Both random error terms, RI and *e*, came from normal distributions with a mean of zero and variances of $\sigma_{g}^{2}$ and $\sigma_{e}^{2}$ (Table 1C). Error variances summed to 10 and were set such that the ICC equaled 0.1, 0.5, or 0.9 (Table 1C). The predictor–response relationship was linear (Eq. 1) or exponential (Eq. 2), where the intercept $\beta_{0}=1$ . There were 72 parameter sets (Table 1C), each of which was used to generate 1,000 datasets. CRSEs were applied to a model fit with $Y=\beta_{0}+\beta_{1}X+\beta_{2}Time+\beta_{3}X\times Time$ . To compare its performance to that of models with incorrect or correct fixed effects, we tested it alongside NLR and LMM. NLR was underparameterized and modeled $Y=\beta_{0}+\beta_{1}X$ , whereas the LMM was fit with the same model as the CRSE and assumed a random intercept. The LMM matched the true data-generating model and would be expected to have the best performance. Estimates and *p*-values were extracted from the results for comparison.

The output from each simulation is available at https://github.com/HallLab/ldasimulations.

#### 2.1.2 Metrics compared in the simulation study

There were 1,000 unique datasets simulated for each possible combination of parameters (Table 1). All methods were applied to the dataset as described previously. Estimates and *p*-values were extracted for any non-intercept β coefficient produced by the model. We then calculated the power/FPR, estimate accuracy, and estimate precision per 1,000-dataset replicate. To determine the power and FPR, we used the standard Wald test output, which tests null hypothesis $H_{0}:\beta =0$ . For terms (i.e., X , Time , and X×Time ) with a true effect, power was the proportion of tests with *p* < 0.05 among the 1,000 replicates. The FPR was the proportion of tests with null effects with *p* < 0.05 among the 1,000 replicates. We considered the FPR to be controlled if within Bradley’s liberal range of 2.5%–7.5% (Bradley, 1978). The NLR, AGG, and CRSE models used *N-p* degrees of freedom, where p is the number of estimated parameters (Bates et al., 2015; Kuznetsova et al., 2017).

To gauge the estimation accuracy and precision, we calculated the difference between the observed and expected effect sizes ( $X_{i}$ ) for each output. Then, for each 1,000-replicate set, we identified the median difference and calculated the median absolute deviation (MAD) of differences. The MAD is defined as $Median\left(\left|X_{i}-\tilde{X}\right|\right)$ , where $\tilde{X}$ is the median difference. The median difference was used to assess the estimate accuracy, while the MAD was used to assess the estimate precision. We chose median-based summary statistics due to large outliers in the observed–expected difference produced by the FE, which complicated plotting. In simulations where NLR and CRSE were fit with the same model, their estimates were identical, so only NLR estimation accuracy and precision were reported. Figures of power, FPR, and estimate summary statistics were created using *ggplot2* (Wickham, 2016) and *viridis* color palettes (Garnier et al., 2023).

### 2.2 Motivated application to a real longitudinal dataset

#### 2.2.1 Dataset and variable descriptions

To demonstrate how the choice of method affects the output, we applied a selection of the aforementioned methods to longitudinal data from a female adolescent cohort (*n* = 460) collected from the catchment area of a Midwest US hospital (Noll et al., 2022). A third of participants had cases of child sexual abuse (CSA) substantiated in the prior year; the remaining were demography- or census-matched controls (Noll et al., 2022). Participants were enrolled at ages 12–16 years and followed for 3 years to assess the health and development between CSA-exposed and -unexposed youths (Noll et al., 2022). Due to the high prevalence of CSA, PTSD was likely to develop among participants (Haag et al., 2022). We chose self-reported PTSD symptoms as our phenotype and examined how symptom development was affected by age, a PTSD-PRS, potentially traumatic events (PTEs), CSA, and income (Table 2). Noll et al. (2022) and Haag et al. (2022) described cohort design and variables in more detail (Haag et al., 2022; Noll et al., 2022).

**TABLE 2:** Variables from the longitudinal cohort examining post-traumatic stress disorder.

Variable	Description	Time-variant
Post-traumatic stress disorder (PTSD) symptoms	Self-report from the Comprehensive Trauma Interview (Shenk et al., 2016). Symptom counts summed for the total score	Yes
PTSD-polygenic risk score (PTSD-PRS)	PRS for PTSD. Separate scores calculated for African-ancestry and European-ancestry strata. Higher scores indicate greater ostensible genetic vulnerability to PTSD	No
Child sexual abuse (CSA)	Participants with CSA were recruited within 1 year of the substantiation of abuse. Unexposed individuals who experienced CSA during the study were excluded from our analysis	No
Income	Ordinal categorical variable denoting household income. Twelve levels	No
Age	Time difference between the date of birth and visit date for data collection	Yes
Potentially Traumatic Event (PTE)	Count of self-reported PTEs from the Comprehensive Trauma Interview (Shenk et al., 2016). At visit 1, the participant reported on all previous PTEs. For visits 2 and 3, the participant reported on any intervening PTEs	Yes

To compute a PTSD-PRS, meta-GWAS summary statistics from the study by Nievergelt et al. (2019) were extracted from stratified African- and European-ancestry analyses (Nievergelt et al., 2019). We kept SNPs (build GRCh37) with a minor allele frequency >1% and imputation quality ≥0.8. We removed SNPs with complementary A1 and A2 alleles and excluded duplicates. The African-ancestry summary statistic data retained 14,051,262 SNPs, while the European-ancestry summary statistic data retained 8,116,466 SNPs.

In our natural cohort, genome-wide SNP data were available for 408 samples genotyped on the Infinium Global Screening Array (build GRCh37). Using PLINK v1.9, we (1) applied a 99% sample call rate; (2) imposed a 99% variant call rate and 1% minor allele frequency; (3) deleted duplicate SNPs; and (4) calculated identity-by-descent to remove related pairs where $\hat{\pi }$ > 0.125. To calculate the identity-by-descent, the data were linkage disequilibrium (LD)-pruned to r^2^ < 0.2 and, among related pairs, individuals with greater variant missingness were removed. To infer genetic ancestry, we combined our cohort with the 2,504 samples from the 1000 Genomes Project (1 KG) Phase 3 release (1000 Genomes Project Consortium et al., 2015). We excluded 110 samples from the 1 KG reference panel in which the IBD met $\hat{\pi }$ > 0.1875. Using PLINK 1.9, the combined variant set was LD-pruned (r^2^ < 0.2), and principal components (PCs) were estimated within the 1 KG reference panel. PCs for our natural dataset were determined by projecting onto the axes determined by the reference panel. The genetic ancestry was inferred using the HARE approach, which uses a support vector machine to predict ancestry and is described by Fang et al. (2019). We used the hare approach to infer African or European ancestry as there were too few individuals of other self-reported race/ethnicities in our natural dataset. The sample was stratified into African and European genetic ancestry subgroups. The genetic data of the cohort were cross-referenced with the meta-GWAS summary statistics to retain SNPs available in both datasets. The African-ancestry stratum included 173 samples and 339,749 SNPs. The European-ancestry stratum included 176 samples and 440,099 SNPs. If necessary, SNPs from our cohort were strand-flipped, and reference alleles were reassigned to match the summary statistics.

SNP coefficient estimates were adjusted in African-ancestry and European-ancestry participants using the PRS with continuous shrinkage (PRS-CS) method developed by Ge et al. (2019) and using the 1000 Genomes LD reference panel, respectively (Ge et al., 2019). The PRS-CS input parameters were as follows: *phi* was 1 × 10^−2^, the African-ancestry sample size was 15,339 participants, and the European-ancestry sample size was 174,659 participants [sample sizes were obtained from the study by Nievergelt et al. (2019)]. The PRS was computed using the score function in PLINK on the adjusted effect sizes from PRS-CS. We estimated ancestry-specific PCs using linkage disequilibrium-pruned (r^2^ < 0.2) data within each ancestral stratum. A total of 20 PCs per stratum were regressed out of their respective PTSD-PRS in R. Residuals from each regression were the final ancestry-specific PTSD-PRSs.

#### 2.2.2 Analytical strategy applied to natural longitudinal data

This application was designed to show how different methods can produce varying results even if applied to the same natural dataset. From our cohort, we selected three waves of data on PTSD symptoms, PTSD-PRS, PTE, CSA, income, and age. We restricted the cohort to individuals of African or European ancestry and required that samples have complete data at all three time points. We excluded initially unexposed participants who experienced CSA during the study. There remained 124 participants in the African-ancestry subsample and 125 participants in the European-ancestry subsample with complete information. The following analyses were performed separately in each stratum defined by genetic ancestry.

We defined a longitudinal model wherein age, PTSD-PRS, PTE, CSA, and income were expected to influence PTSD symptoms over time. We also specified the interaction between age and all other predictors to allow the variables to show time-varying effects. PTE, PTSD-PRS, and income were z-scored before fitting the model. For individual *i* at time *t*, the model tested was ${PTSD}_{it}={Age}_{it}+{PRS}_{i}+{CSA}_{i}+{PTE}_{it}+{Income}_{i}+{PRS}_{i}\times {Age}_{it}+{CSA}_{i}\times {Age}_{it}{+PTE}_{it}\times {Age}_{it\;}+{Income}_{i}\times {Age}_{it}$ . Model terms were significant if the *p*-value from the two-sided significance test $H_{0}:\beta =0$ was less than 0.05. We fit this model using three methods—LMM, GEE, and naïve regression adjusted with CRSEs.

The LMM was fit using *lme4* and *lmerTest* (Bates et al., 2015; Kuznetsova et al., 2017). Prior to fitting, we tested unconditional means and unconditional growth models to determine whether the LMM should contain random intercepts and/or slopes. The unconditional growth model failed to converge; this did not improve when the *lme4* optimizer algorithm was changed. Thus, we only specified a random intercept. We fit the GEE using *geepack* and specified an “exchangeable” working correlation structure (Halekoh et al., 2006). CRSEs were applied with *lmtest* and the cluster-robust variance correction from the *sandwich* package (Zeileis and Hothorn, 2002; Zeileis, 2004; Zeileis, 2006; Zeileis et al., 2020). CRSEs used the default degrees of freedom and the HC1 correction.

Lastly, we averaged the select variables across all time points and fit an AGG model; as CSA, PRS, and income were constant over time, only PTSD symptoms and PTE had to be averaged. As the prior methods all fit models specifying development over time (age and its interactions), the AGG model served to compare how removing possible change-over-time from the data affects results. Likewise, we used complete data across all three time points. The average PTE, PTSD-PRS, and income were z-scored. The AGG model fit was ${\bar{PTSD}}_{i}={PRS}_{i}+{CSA}_{i}+{\bar{PTE}}_{i}+{Income}_{i}$ . Model terms were significant if *p* < 0.05 in a two-sided significance test ( $H_{0}:\beta =0$ ). The AGG model used the R *stats* general linear model function.

This application of LMMs, GEEs, CRSEs, and AGG to a natural dataset is intended to demonstrate how the method choice affects the interpretation of results and potentially any follow-up analyses based on the findings. We did not use a multiple test correction because the aim was to compare changes in output and because the models were inherently non-independent as they were fit using the same data and most or all of the same variables. We caution against using the results from this application to make strong claims about genetic/environmental influences on PTSD development, especially since models including CSA and PTE may need to include other potential environmental and psychosocial confounders (Keane et al., 2006; Qi et al., 2016; Shalev et al., 2017).

## 3 Results

### 3.1 NLR or CRSE had the highest power to detect the effect of X in simulated data with time-varying effects

We simulated longitudinal datasets where the response trajectory was dependent on X , Time , and the interaction X×Time . This represents data where the predictor of interest, X , affects the change of phenotype Y over time via the X×Time effect. In such a case, methods that ignore the effects of Time or X×Time may produce improper results. We tested the NLR, CRSE, AGG, FE, LMM, and GEE approaches on these data. The NLR, CRSE, and AGG approaches all disregarded Time and X×Time as terms in the model. The remaining methods modeled the three response-generating predictors X , Time , and X×Time .

**FIGURE 1:**
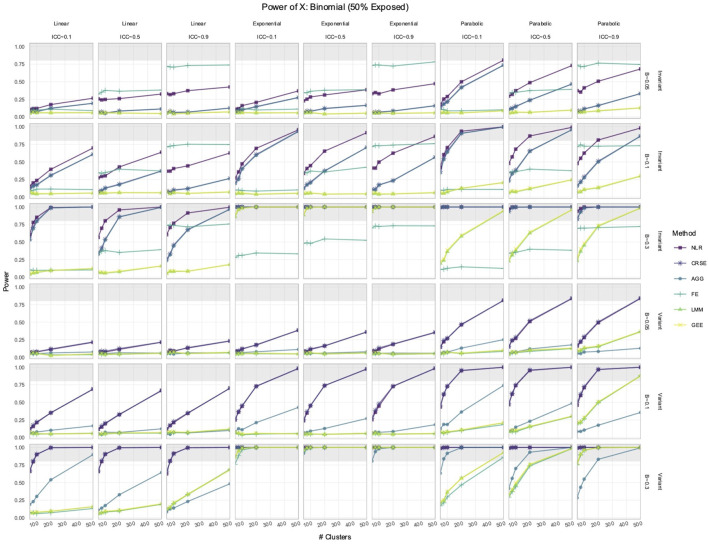
Power to detect the effect of the $Bin\left(n,0.5\right)$ predictor. The *x*-axis indicates the number of clusters. The *y*-axis indicates the power. The shaded gray region indicates where power reaches or exceeds 80%. The panel columns correspond to the simulation linearity and the intraclass correlation coefficient (ICC) of the data. The panel rows correspond to whether the predictor was time-variant and the true effect size (*β*). Each method has a power trajectory, color-coded according to the legend. NLR, naïve linear regression; CRSE, cluster-robust standard error; AGG, aggregate regression; FE, fixed effects; LMM, linear mixed model; GEE, generalized estimating equation.

**FIGURE 2:**
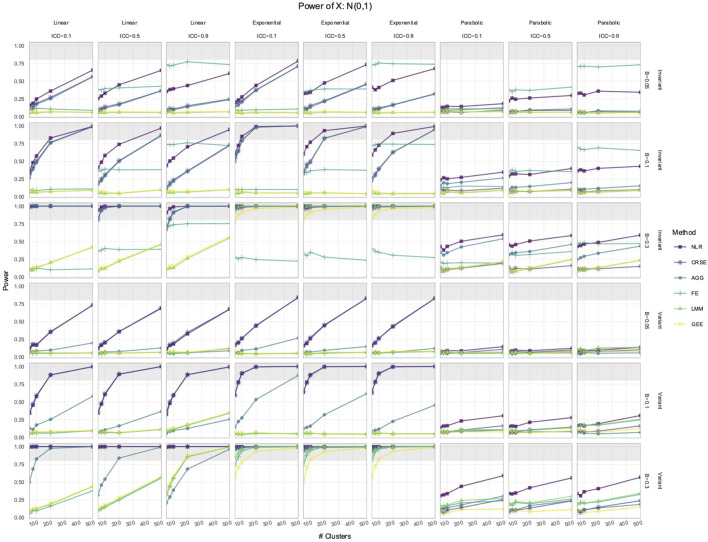
Power to detect the effect of an $N\left(0,1\right)$ predictor. The *x*-axis indicates the number of clusters. The *y*-axis indicates the power. The shaded gray region indicates where power reaches or exceeds 80%. The panel columns correspond to the simulation linearity and the ICC of the data. The panel rows correspond to whether the predictor was time-variant and the true effect size (*β*). Each method has a power trajectory, color-coded according to the legend. NLR, naïve linear regression; CRSE, cluster-robust standard error; AGG, aggregate regression; FE, fixed effects; LMM, linear mixed model; GEE, generalized estimating equation.

Out of all the methods, the NLR usually had the highest power to detect the effect of X , while LMM and GEE had the lowest (Figures 1, 2). However, the relative performance of methods regarding the power to detect X depended on (1) whether X was time-invariant (fixed across time) or time-variant (varying across time); (2) the strength of dependency, as defined by the ICC; and (3) if the predictor–response relationship was parabolic. When the predictor X was time-invariant, then NLR had the highest power. The CRSE and AGG were equivalent regarding power but had less power than NLR. The lowest power was that of LMM and GEE, which also had power comparable to each other. The FE model did not have a set performance rank; its power solely depended on the ICC, and in simulations where ICC = 0.9, it sometimes achieved the highest power of all the methods, surpassing even NLR. In simulations with a time-variant X , NLR and CRSE had the highest power of all the methods. AGG had the second highest power. The FE, LMM, and GEE approaches had, equivalently, the lowest power. An exception to these trends in power occurred in simulations with a standard normal X and parabolic predictor–response relationship (Figure 2). In time-invariant simulations, the power ranking was (1) NLR or FE, (2) AGG, and (3) LMM, GEE, and CRSE. In time-variant simulations, NLR had the highest power, followed by the LMM and FE, and then the AGG, GEE, and CRSE approaches. A notable feature of each method was their sensitivity to the ICC. The AGG, FE, LMM, and GEE all displayed consistent trends between power and the ICC. The power of the AGG model decreased as the ICC increased. In contrast, FE, LMM, and GEE gained power with the increase in the ICC. The CRSE power decreased with the ICC in time-invariant simulations, where its power trajectory was identical to that of AGG. However, there was no trend between CRSE power and the ICC in time-variant simulations.

### 3.2 FE, LMM, and GEE had comparable power to detect effects of Time and *X* × Time in simulated data with time-varying effects

**FIGURE 3:**
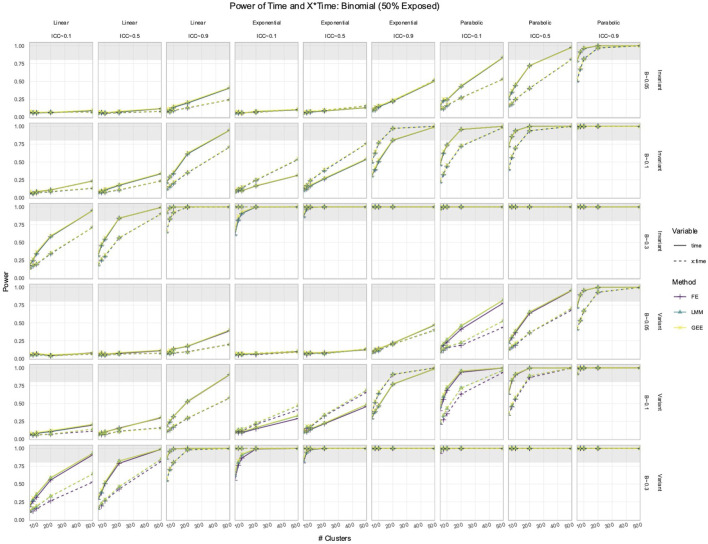
Power to detect the effect of Time and X×Time in simulations with a $Bin\left(n,0.5\right)$ predictor. The *x*-axis indicates the number of clusters. The *y*-axis indicates the power. The shaded gray region indicates where power reaches or exceeds 80%. The panel columns correspond to the simulation linearity and the ICC of the data. The panel rows correspond to whether the predictor was time-variant and the true effect size (*β*). Each method has a power trajectory, color-coded according to the legend. Solid lines denote the power of Time , while dashed lines denote the power of X×Time . FE, fixed effects; LMM, linear mixed model; GEE, generalized estimating equation.

**FIGURE 4:**
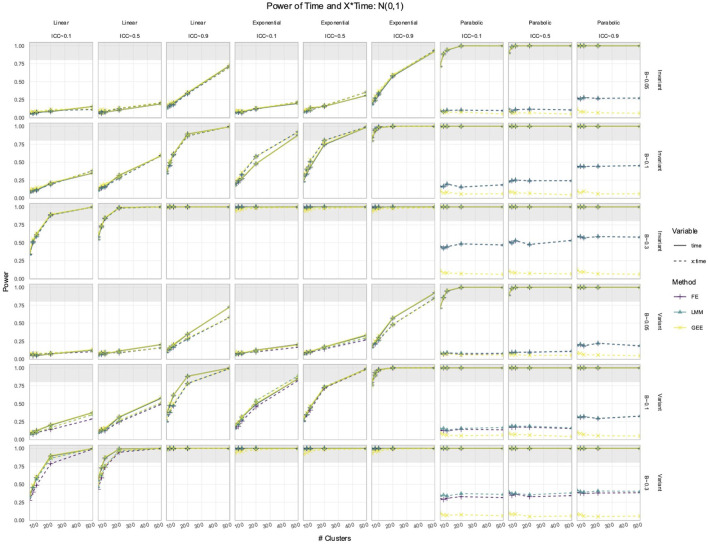
Power to detect the effect of Time and X×Time in simulations with a $N\left(0,1\right)$ predictor. The *x*-axis indicates the number of clusters. The *y*-axis indicates the power. The shaded gray region indicates where power reaches or exceeds 80%. The panel columns correspond to the simulation linearity and the ICC of the data. The panel rows correspond to whether the predictor was time-variant and the true effect size (*β*). Each method has a power trajectory, color-coded according to the legend. Solid lines denote the power of Time , while dashed lines denote the power of X×Time . FE, fixed effects; LMM, linear mixed model; GEE, generalized estimating equation.

In the simulated longitudinal datasets, where the response trajectory was dependent on X , Time , and the interaction X×Time , only the LMM, GEE, and FE explicitly modeled Time and an X×Time interaction. Their power to detect the effects of Time or X×Time was largely identical (Figures 3, 4), except in parabolic simulations with a standard normal predictor, where a large performance difference emerged (Figure 4). The other deviations from their comparable performance were as follows: (1) FE had slightly less power than the LMM and GEE in time-variant simulations with low ICCs and (2) GEE was the slowest to reach 100% power in exponential simulations. However, in parabolic simulations with a standard normal X , the power to detect the effect of X×Time differed between the FE, LMM, and GEE (Figure 4). The LMM and FE had the highest and equivalent power, whereas the GEE power remained around 10% for all simulations. Each method exhibited a positive association between the power to detect Time or X×Time and the ICC.

### 3.3 NLR and AGG always had a biased estimation of *X* in simulated data with time-varying effects

While power determines whether a true signal can be discovered by a method, estimation accuracy and precision reveal whether the reported magnitude of the signal is reliable. Overestimation or underestimation of an effect can portray an unrealistic relationship between the predictor and phenotype. We calculated the difference between each estimated and true effect. A positive difference indicated that the estimated β was greater than the actual β , and the effect was overestimated. The reverse was true if the difference was negative. From this distribution of estimate differences, we obtained the median difference and calculated the MAD of the differences to compare the method accuracy and precision, respectively. As the NLR and CRSE had the same coefficient estimates, only NLR results are reported.

**FIGURE 5:**
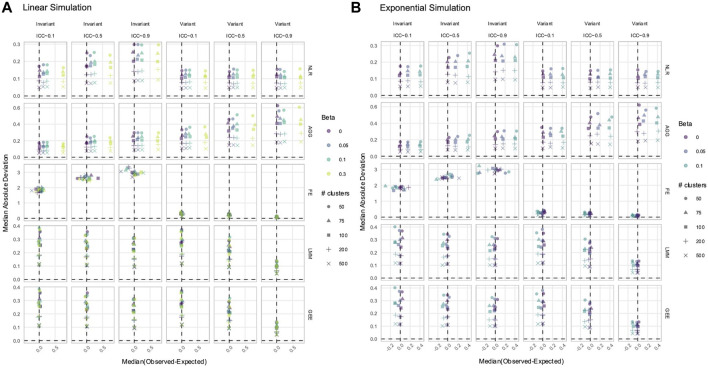
Estimation of the $N\left(0,1\right)$ predictor in linear **(A)** and exponential **(B)** simulations. The *x*-axis indicates the median estimate difference. The *y*-axis indicates the median absolute deviation (MAD); the *y*-axis range varies by panel. Point color and shape represent the effect size (*β*) and the number of clusters, respectively. For the exponential simulation **(B)**, an effect size of 0.3 is excluded. The dashed vertical and horizontal lines indicate a median and MAD of zero, respectively. Panel columns correspond to predictor time-variance and the ICC. Methods are plotted along panel rows. NLR, naïve linear regression; AGG, aggregate regression; FE, fixed effects, LMM, linear mixed model; GEE, generalized estimating equation.

**FIGURE 6:**
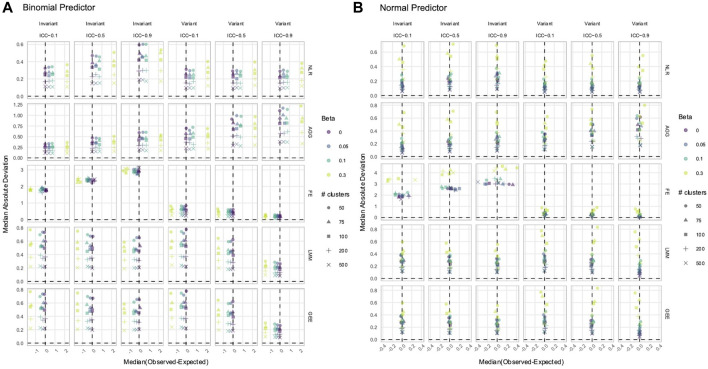
Estimation of the **(A)** $Bin\left(n,0.5\right)$ or **(B)** $N\left(0,1\right)$ predictor in parabolic simulations. The *x*-axis indicates the median estimate difference. The *y*-axis indicates the MAD; the *y*-axis range varies by panel. Point color and shape represent the effect size (*β*) and the number of clusters, respectively. The dashed vertical and horizontal lines indicate a median and MAD of zero, respectively. Panel columns correspond to predictor time-variance and the ICC. Methods are plotted along panel rows. NLR, naïve linear regression; AGG aggregate regression; FE, fixed effects; LMM, linear mixed model; GEE, generalized estimating equation.

In data with time-varying effects, where the response trajectory is partly determined by the effect of X×Time , longitudinal methods that ignore the effects of Time and X×Time may produce biased coefficient estimates. Estimate bias only occurred if the true effect was non-null, and bias worsened as the effect size increased (Figures 5, 6; Supplementary Figures S2, S3). We found that when the NLR and AGG were fit without modeling Time and X×Time , they routinely overestimated the effect of X to about the same degree (Figures 5, 6; Supplementary Figures S2, S3). Neither did the FE, LMM, and GEE produce biased estimates in simulations with a linear predictor–response relationship (Figure 5A; Supplementary Figure S2A), nor did any methods exhibit bias if the simulated data had a normally distributed X and a parabolic predictor–response relationship (Figure 6B). However, all methods were biased in the remaining exponential or parabolic simulations, with the NLR and AGG having larger bias than the FE, LMM, and GEE (Figures 5B, 6B; Supplementary Figures S2B, S3). The NLR, LMM, and GEE had the greatest estimate precision (the lowest MAD). In time-invariant simulations, FE was least precise, having the highest MAD. The AGG method had the highest MAD in time-variant simulations. The LMM and GEE always had a negative association between the MAD and ICC; as the ICC increased, their estimation precision improved. This negative association between the MAD and ICC was only exhibited by FE in time-variant simulations.

### 3.4 FE, LMM, and GEE only had a biased estimation of Time and *X* × Time in nonlinear simulated data with time-varying effects

**FIGURE 7:**
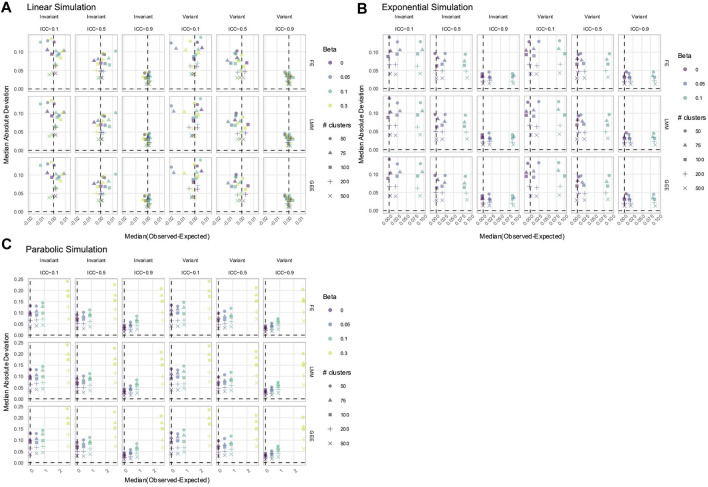
Estimation of Time in **(A)** linear **(B)** exponential, and **(C)** parabolic simulations with an $N\left(0,1\right)$ predictor. The *x*-axis indicates the median estimate difference. The *y*-axis indicates the MAD; the *y*-axis range varies by panel. Point color and shape represent the effect size (*β*) and the number of clusters, respectively. For the exponential simulation **(B)**, an effect size of 0.3 is excluded. The dashed vertical and horizontal lines indicate a median and MAD of zero, respectively. Panel columns correspond to predictor time-variance and the ICC. Methods are plotted along panel rows. FE, fixed effects; LMM, linear mixed model; GEE, generalized estimating equation.

To estimate true time-varying effects, the terms Time and X×Time must be fit by the applied model. In our simulated datasets with time-varying effects, the methods that modeled effects were the FE, LMM, and GEE. They had almost identical values and trends of the median estimate difference and MAD for Time (Figure 7; Supplementary Figures S4, S5) and X×Time (Supplementary Figures S6–S8) effects. In linear simulations, they produced unbiased estimates of Time and X×Time , but their estimation was inaccurate if the predictor–response relationship was exponential or parabolic. Each had a MAD that decreased as the ICC increased, showing improvements in the estimate precision as the strength of dependency increased.

### 3.5 If *X* was time-invariant, NLR and FE had inflated FPRs in simulated data with time-varying effects

**FIGURE 8:**
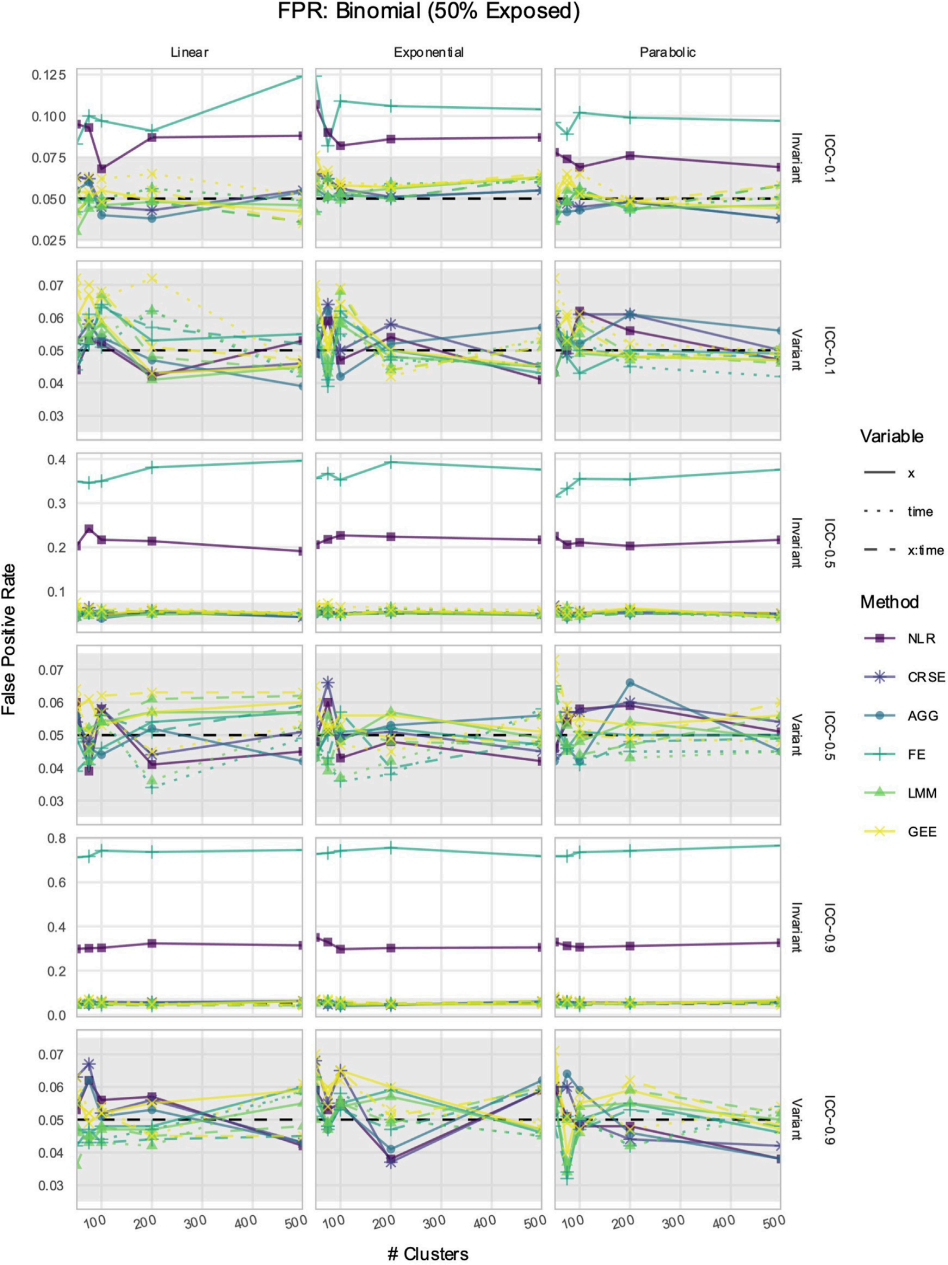
False positive rate (FPR) in simulations with a $Bin\left(n,0.5\right)$ predictor. The *x*-axis indicates the number of clusters. The *y*-axis indicates the FPR. The shaded gray region is Bradley’s liberal FPR region from 2.5% to 7.5%. The panel columns correspond to the simulation linearity. The panel rows correspond to the ICC and the predictor time-variance. Each method has an FPR trajectory, color-coded according to the legend. The line type denotes the variable. NLR, naïve linear regression; CRSE, cluster-robust standard error; AGG, aggregate regression; FE, fixed effects; LMM, linear mixed model; GEE, generalized estimating equation.

The FPR was considered maintained if it lay within Bradley’s liberal range of 2.5%–7.5% (Bradley, 1978). In time-invariant simulations, the NLR and FE models had inflated FPRs when detecting the effect of X ; FE had higher inflation than NLR (Figure 8; Supplementary Figure S9). Their FPR inflation increased with the ICC. Neither method had elevated FPRs when the predictor X was time-variant. The GEE and CRSE occasionally had FPRs above 7.5% when the number of clusters was small (*i* ≤ 100) but not to the degree of the NLR and FE inflation.

### 3.6 All methods had unbiased estimation of X in simulated data with no time-varying effects

In some longitudinal data, the effect of a predictor X does not affect the change of the phenotype trajectory. This means that X does not have any time-varying effects, as defined by X×Time , and that methods that do not explicitly model this interaction may no longer exhibit estimate bias when estimating X . We generated longitudinal data without time-varying effects and compared the NLR, AGG, and CRSE—which modeled the response regressed on X alone—against a correctly specified LMM (modeling the main effects of and Time ) and an incorrectly specified LMM that contained an X×Time interaction (LMM+Int).

**FIGURE 9:**
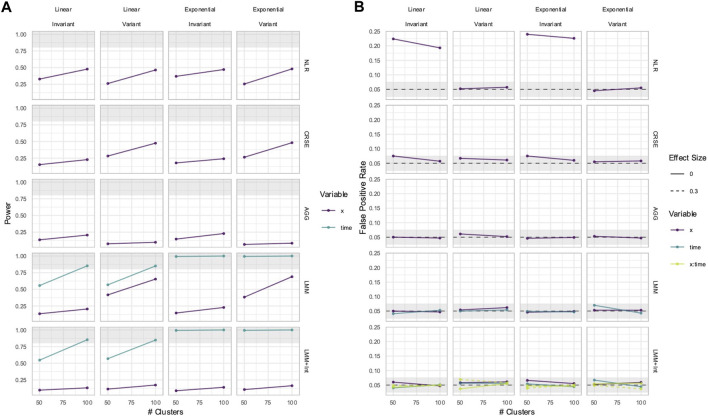
Power **(A)** and FPRs **(B)** in simulations without an X×Time interaction. The *x*-axis denotes the number of clusters. The *y*-axis indicates the **(A)** power or **(B)** FPR. On power plots, the shaded region is where power reaches or exceeds 80%. The shaded region on FPR plots is Bradley’s liberal FPR region from 2.5% to 7.5%. The panel columns correspond to the simulation linearity and the predictor time-variance. The panel rows correspond to the method. The line color refers to the variables in the model. For FPR plots only, the line type reflects the effect size. NLR, naïve linear regression; CRSE, cluster-robust standard error; AGG, aggregate regression; LMM, linear mixed model; LMM+Int, overparameterized LMM with an X×Time interaction.

**FIGURE 10:**
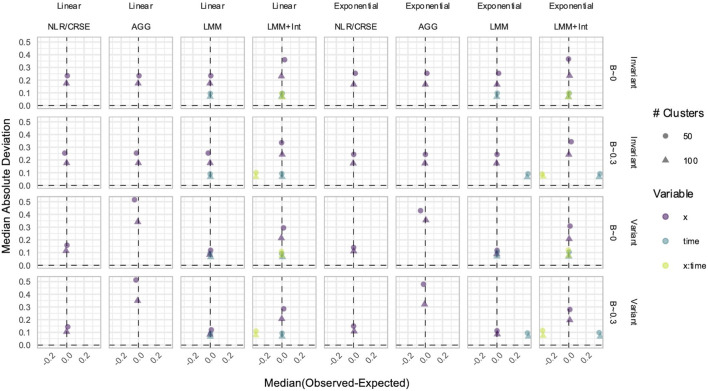
Estimation in simulations without an X×Time interaction. The *x*-axis indicates the median estimate difference. The *y*-axis indicates the MAD; the *y*-axis range varies by panel. Point color and shape represent the effect size (*β*) and the number of clusters, respectively. The dashed vertical and horizontal lines indicate a median and MAD of zero, respectively. Panel columns denote simulation linearity and the method. Panel rows correspond to predictor time-variance and effect size (*β*). NLR, naïve linear regression; CRSE, cluster-robust standard error; AGG, aggregate regression; LMM, linear mixed model; LMM+Int, overparameterized LMM with an X×Time interaction.

The power to detect the effect of the predictor X was not affected by whether the predictor–response relationship was linear or exponential (Figure 9A). When the simulated X was time-invariant, then NLR had the highest power, overparameterized LMM+Int had the lowest power, and the CRSE, AGG, and LMM had comparable, intermediate power. However, when X was time-variant, the LMM had the highest power, CRSE and NLR methods had intermediate power, and LMM+Int and AGG methods had the lowest power. Only LMM and LMM+Int explicitly modeled Time , and they exhibited equal power to detect its effect (Figure 9A). In time-invariant simulations, the FPR of NLR was inflated to approximately 20% (Figure 9B). The CRSE had a slightly increased FPR in time-invariant simulations but did not exceed the 7.5% upper limit of Bradley’s liberal range. All other methods controlled the FPR, for both the effect of X and of Time , where applicable. The LMM+Int approach incorrectly modeled X×Time but kept the FPR at the nominal rate, regardless of whether X and Time had a true individual effect. In terms of estimation accuracy, no method was biased when estimating the effect of X (Figure 10). However, LMM+Int and AGG models had lower estimate precision (higher MAD) than NLR, CRSE, and LMM. Both LMM and LMM+Int had biased estimates of Time and X×Time , if applicable, in non-null exponential simulations (Figure 10). LMM+Int had lower precision than LMM when estimating the effects of Time and X×Time .

### 3.7 CRSE had no estimate bias if all relevant predictors were included in the model

CRSEs can be applied atop any regression model as long as the clustering is two-level and group membership is known. Previously, our CRSE approach ignored time and was applied to a model with the form $Y=\beta_{0}+\beta_{1}X$ . Nevertheless, CRSEs can also be applied to models that do not consider time as “nuisance,” which would no longer disadvantage it when analyzing data with time-varying effects. We simulated longitudinal data with time-varying effects (a true X×Time interaction) and applied CRSEs to a model fit with all data-generating terms: $Y=\beta_{0}+\beta_{1}X+\beta_{2}Time+\beta_{3}X\times Time$ . The performance of the CRSE was compared to an underparameterized NLR, which regressed the response only against X , and a correctly specified LMM.

**FIGURE 11:**
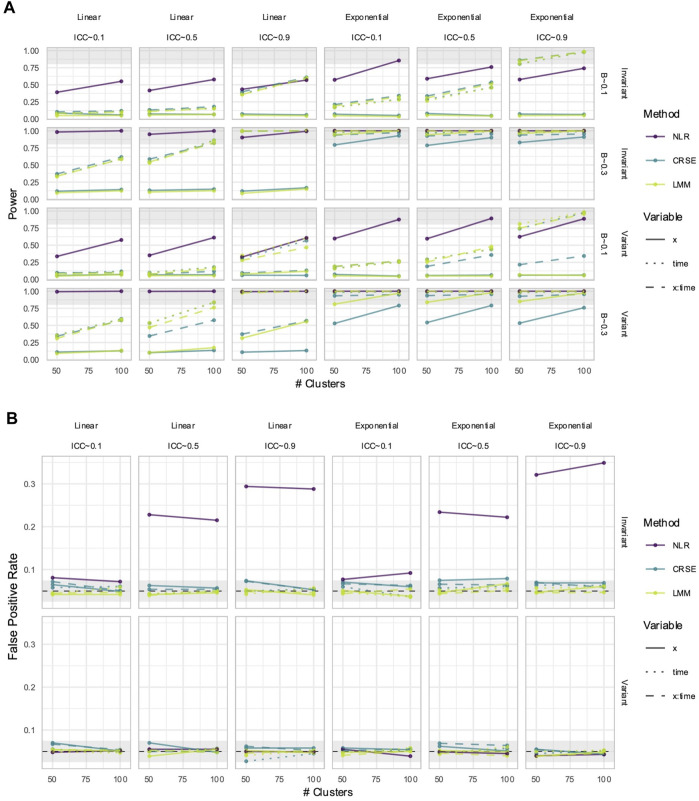
Power **(A)** and false positive rates (FPRs) **(B)** in simulations with correctly specified CRSE model. The x-axis number of clusters. The y-axis indicates **(A)** power or **(B)** FPR. On power plots, the shaded region is where power reaches or exceeds 80%. The shaded region on FPR plots is Bradley's liberal FPR region from 2.5% – 7.5%. The panel columns correspond to the simulation's linearity and the intraclass correlation coefficient (ICC). The panel rows correspond to the predictor's time-variance and, on power plots only, the effect size (β). Line color and type denote method and variable, respectively. NLR=naïve linear regression, CRSE=cluster-robust standard error, LMM= linear mixed model.

**FIGURE 12:**
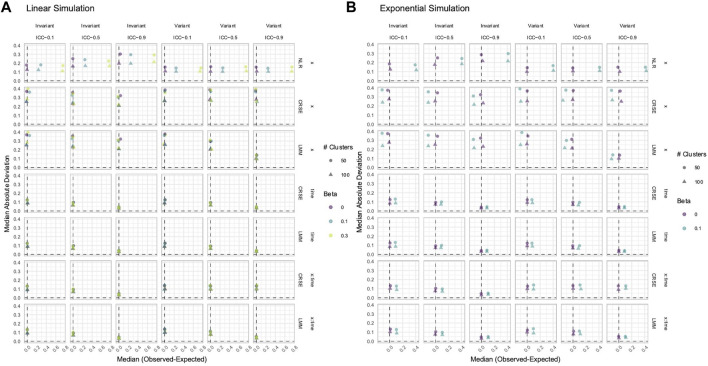
Estimation in **(A)** linear or **(B)** exponential simulations with a correctly specified CRSE model. The x-axis indicates median estimate difference. The y-axis indicates median absolute deviation (MAD); the y-axis range varies by panel. Point color and shape represent effect size (β) and the number of clusters, respectively. For the exponential simulation **(B)**, the effect size of 0.3 is excluded. Point shape is the number of clusters. The dashed vertical and horizontal lines indicate a median or MAD of zero, respectively. Panel columns denote the simulation linearity and intraclass correlation coefficient (ICC). Panel rows correspond to the variable and method. NLR=naïve linear regression, CRSE=cluster-robust standard error, LMM= linear mixed model

NLR had higher power to detect X than the CRSE and LMM (Figure 11A). In time-invariant simulations with a linear predictor–response relationship, LMM and CRSE power was comparable. However, in the remaining simulations, LMM power exceeded that of the CRSE, especially as the ICC increased. The NLR had an inflated FPR when the predictor was time-invariant, which increased as the ICC increased (Figure 11B). Neither the CRSE nor LMM had FPR inflation, although the CRSE had a slightly higher FPR than the LMM (Figure 11B). In simulations with a linear predictor–response relationship, only NLR overestimated the effect of X when there was a true effect (Figure 12A). The LMM and CRSE were unbiased in their estimation of X , Time , and X×Time . All methods were biased in exponential simulations, although NLR had the greatest bias (Figure 12B; Supplementary Figure S10). The MAD of the three methods was within the same range (Figure 12). The CRSE and LMM had an almost identical MAD. The MAD of the LMM showed greater improvement than that of the CRSE with increasing strength of dependency.

### 3.8 Results varied among methods applied to natural data

Among African-ancestry participants, the directions of effects were consistent for all predictors across methods, but statistical significance differed (Table 3). The LMM and GEE had the most similar coefficients. The LMM and CRSE methods found positive associations between age and PTSD symptoms ( $\beta_{LMM}$ = 0.593, $p_{LMM}$ = 0.006; $\beta_{CRSE}$ = 0.593, $p_{CRSE}$ = 0.005). No terms were significant in GEE. The AGG model found that exposure to CSA ( $\beta_{AGG}$ = 2.498, $p_{AGG}$ = 1.07 × 10^−4^) and average PTE ( $\beta_{AGG}$ = 3.69, $p_{AGG}$ = 1.33 × 10^−6^) were both associated with greater average PTSD symptoms.

**TABLE 3:** Results of models applied to the African-ancestry or European-ancestry participants.

Term	Estimate				*p*-value			
	LMM	GEE	CRSE	AGG	LMM	GEE	CRSE	AGG
African-ancestry models								
Age	0.593	0.775	0.593		0.006**	0.135	0.005**	
PRS	1.139	1.12	0.67	0.41	0.619	0.59	0.744	0.152
CSA	5.495	5.433	3.895	2.498	0.26	0.302	0.471	1.07 × 10^−4^**
PTE	2.882	2.905	3.69	1.558	0.164	0.21	0.138	1.33 × 10^−6^**
Income	3.077	3.042	2.181	0.545	0.239	0.229	0.401	0.077
PRS × age	−0.043	−0.042	−0.013		0.768	0.762	0.926	
CSA × age	−0.186	−0.182	−0.089		0.549	0.599	0.803	
PTE × age	−0.078	−0.079	−0.12		0.562	0.613	0.474	
Income × age	−0.163	−0.16	−0.105		0.321	0.331	0.535	
European-ancestry models								
Age	0.314	0.286	0.516		0.069	0.579	0.007**	
PRS	4.401	4.382	3.8	0.178	0.049*	0.031*	0.101	0.536
CSA	2.444	2.481	4.52	2.075	0.656	0.666	0.524	0.007**
PTE	−1.941	−1.887	0.826	2.002	0.375	0.526	0.753	3.55 × 10^−8^**
Income	0.115	0.171	2.274	0.095	0.962	0.939	0.386	0.771
PRS × age	−0.282	−0.281	−0.242		0.056	0.038*	0.116	
CSA × age	0.037	0.034	−0.124		0.917	0.929	0.791	
PTE × age	0.207	0.204	0.057		0.147	0.283	0.739	
Income × age	−0.027	−0.03	−0.161		0.867	0.834	0.346	

LMM, linear mixed model; GEE, generalized estimating equation; AGG, aggregate regression; CRSE, cluster-robust standard error; PRS, polygenic risk score; CSA, child sexual abuse; PTE, potentially traumatic event. *p < 0.05 and **p < 0.01.

Differing directions of effect were observed when comparing the PTE and CSA × age results in European-ancestry samples, although the LMM and GEE estimates remained the most similar (Table 3). In the LMM and GEE, the coefficient for PTEs was negative, whereas for the CRSE and AGG, it was positive. For the CSA × age interaction, the LMM and GEE estimated a positive coefficient, implying that the effect of CSA increases with age, whereas the CRSE approach found a negative interaction. However, neither the PTE nor CSA × age was significant in any model. Rather, each approach found a different subset of statistically significant terms when applied in European-ancestry participants (Table 3). The LMM found a significant positive association of PRSs with PTSD symptoms ( $\beta_{LMM}$ = 4.401, $p_{LMM}$ = 0.049). Both the PRS ( $\beta_{GEE}$ = 4.382, $p_{GEE}$ = 0.031) and PRS × age ( $\beta_{GEE}$ = −0.281, $p_{GEE}$ = 0.038) were significant in the GEE model. The association between PTSD symptoms and age was positive when CRSEs were applied ( $\beta_{CRSE}$ = 0.516, $p_{CRSE}$ = 0.007). The AGG method found significant positive associations between PTSD symptoms and CSA ( $\beta_{AGG}$ = 2.076, $p_{AGG}$ = 0.007) and PTE ( $\beta_{AGG}$ = 2.002, $p_{AGG}$ = 3.55 × 10^−8^) risk factors.

## 4 Discussion

Longitudinal data provide researchers an avenue to investigate and understand how risks or buffers impact the development of disease. However, data with repeated measures on individual samples are implicitly dependent. This violates the independence assumption of linear or logistic regression models primarily used in GWASs or PRSs. Evaluating the effect of genetic and environmental risk factors on a repeatedly measured phenotype requires a statistical methodology that accommodates dependency. Many such methods exist, but not all may be suitable in a given analysis. Therefore, we examined five LDA methods and compared their performance among each other and to a naïve estimator. Of these methods, the LMM and GEE are often recommended for the analysis of dependent data (Gibbons et al., 2010; Garcia and Marder, 2017; McNeish et al., 2017; Woodard, 2017). We also considered AGG, CRSE, and FE approaches, which accommodate dependency, and NLR, which does not. Each method was applied to simulated longitudinal datasets to compare estimation, power, and FPR. Methods were further implemented in a cohort of African and European ancestry examining PTSD symptoms in maltreated adolescents to show how the method choice impacts the interpretation of polygenic and environmental risk effects on the symptom trajectory.

The results from the simulation suggest that three factors are most crucial to consider when selecting a model for LDA: whether (1) predictor(s) vary across time; (2) the effect of predictor(s) vary over time—i.e., interacts with Time ; and (3) Time is an important experimental variable. The results showed that when the predictor was time-invariant, as expected of genetic risk, NLR or FE was unviable due to FPR inflation. Although null estimates were unbiased, the FPR elevation worsened with increasing dependency within the data (Figure 8). Other methods—CRSE, AGG, LMM, or GEE—prevented FPR inflation when the predictor was time-invariant and should, therefore, be preferred. NLR and FE did not have an inflated FPR when the predictor was time-variant, and in such a case, the power advantage of NLR may be attractive to researchers. However, this depends on whether the predictor interacted with time—if so, then NLR produced biased estimates as it could not model the trajectory change over time (Figure 5A), and if not, then other methods had superior or comparable power (Figure 9A). Furthermore, this point is likely moot when analyzing genetic data, which are time-invariant.

When the effect of the predictor varied over time, any method that did not explicitly model Time —as did NLR, AGG, and usually, CRSE—produced biased estimates (Figure 5A). When the predictor and time were independent, these methods were unbiased, regardless of whether Time was linearly related to the response (Figure 10). NLR still had an increased FPR when the predictor was time-invariant, but AGG, CRSE, and a correctly specified LMM had comparable power without compromising the FPR (Figure 9). If the predictor was time-variant, then the correctly specified LMM had superior power. Overparameterizing the LMM with an X×Time interaction (LMM+Int) did not bias estimates or affect the FPR, but it did diminish the power to detect the effect of X . If there is an interaction between the predictor X and Time , then Time cannot be omitted from the model. If no such interactions exist with the relevant predictor(s), a researcher could consider Time as “nuisance” and opt for CRSEs applied to a regression not modeling Time or use a correctly specified LMM if the effect of Time is of interest (Figures 9, 10). NLR or AGG was not preferred as NLR had FPR inflation when the predictor was time-invariant (Figure 9B), while AGG had greatly reduced power when the predictor was time-variant (Figure 9A). As the AGG approach has limitations when modeling time-variant predictors, it may seem to have applicability in genetic data, which are fixed over time. AGG could be applied if all covariates are time-invariant, none have suspected interactions with Time , and the ICC is low to moderate. If not, the AGG will produce biased estimates or be underpowered relative to other approaches.

The CRSE approach was initially implemented on a linear regression that did not model Time . However, CRSEs can be applied to more complex models that specify Time and time-varying effects. We found that implementing the CRSE atop a regression with correctly specified fixed effects eliminated estimation bias incurred by ignoring time-varying effects (Figure 12A). However, in this scenario, the CRSE approach still performed less well than the LMM as the latter received boosts in power and estimate precision as the ICC increased (Figures 11A, 12A). However, CRSEs could still be a viable option if the researcher has concerns about specifying proper random effects in an LMM or if the dependency is low. However, the CRSE occasionally exceeded Bradley’s liberal range for the FPR when there were less than 100 clusters (Figure 11B; Supplementary Figure S9). Despite performing similarly to the LMM, the GEE also showed this drawback with low cluster numbers (Figure 8; Supplementary Figure S9). Therefore, we recommend LMMs over these methods if sample size is of concern. Otherwise, GEEs could be used interchangeably with LMMs and may be preferred if LMM assumptions are under question.

In simulations with a nonlinear—exponential or parabolic—predictor–response relationship, all methods tended to have heightened power but consistent overestimation or underestimation of true effects. Uniquely, the power of GEE stagnated or even decreased in nonlinear simulations. Given its biased estimates, this power loss may counterintuitively benefit researchers as the inaccurate results of GEE are less likely to reach statistical significance. The overestimation of true effects did not bias the FPR upward in nonlinear simulations. However, researchers should investigate their data prior to analysis to check the linearity assumption. If nonlinearity is evident, then researchers should expect exaggerated coefficient estimates, which may overstate the relationship between risk factors and health outcomes.

We demonstrated how the choice of method impacts the obtained results by applying four longitudinal analysis methods to a natural cohort studying the risk of PTSD. We modeled four predictors and their time-varying effects with LMMs, GEEs, and CRSEs. In applying the AGG approach, we averaged all time-varying variables and fit a model that ignored potential PTSD development over time. Furthermore, our cohort was restricted to individuals with complete data on PTSD, CSA, PTE, PTSD-PRS, and income across all three time points. Thus, each method was applied to the exact same dataset. The LMM, GEE, and CRSE modeled the same fixed effects on the same data but had discrepancies in their results. Despite the LMM and GEE having the most similar estimates, each of these models called a different subset of terms significant across ancestry groups (Table 3). CRSE results had further estimates from those resulting from LMM and GEE models but more consistent statistical significance across ancestral strata. The AGG model, which regressed the average PTSD symptom count on the average predictor values, predictably had the most distinct results. Unlike the other approaches, it found strong significant effects of CSA and PTE on increased average PTSD symptoms (Table 3).

If different researchers had independently applied each of these approaches to our cohort, each would come away with a different interpretation of the role these risk factors play in PTSD. Had LMMs been applied, the results would have suggested that age increases PTSD symptoms among African-ancestry individuals, while the PRS is implicated among European-ancestry individuals. If GEEs were the chosen method, then the PRS and PRS × age would have been implicated in PTSD development only within the European-ancestry subsample. The application of CRSEs would have shown that both African-ancestry and European-ancestry participants showed increased PTSD symptoms with increased age. Lastly, if the researcher had chosen to average all time-varying variables prior to regression, they would have determined that more PTEs on average and CSA exposure led to an increased average PTSD symptom count in both ancestral groups. We are not in the position to state which model is the most appropriate as all methods applied to the cohort accommodated for the within-individual dependency of repeated measures data, and it is unknown whether the data truly meet the assumptions of each method. Nevertheless, we showed that the choice of method has downstream implications as different variables would be highlighted for follow-up investigations, dependent on the approach utilized. Discrepancies among results would be attenuated by acquiring a well-powered sample as our simulations imply that once the sample size, true effect size, and/or intraclass correlation are high enough, all methods will reach maximum power. We previously highlighted three issues researchers should consider when choosing methods: the variability of predictors across time, if the effects of predictors change over time, and whether the research question considers time an important factor. These considerations can guide methodological choices, as can other observations, such as the poorer FPR of GEEs, in small samples or the advantage both LMM and GEE show when within-individual dependency is very high.

Our simulation could not cover all possible data-generating scenarios. We adopted a simple two-level structure consisting of a cluster-level random intercept to simulate dependence and individual residual error. By doing so, our data-generating model met the assumptions of a random intercept LMM. Most of the LMMs we tested in the simulation study matched the data-generating model perfectly, and therefore, the good performance of the LMM across simulations was expected. The results from our simulation suggest that the LMM approach is the most robust method as it controlled the FPR and estimate bias across all samples sizes and had improved precision with increasing ICC values. However, we emphasize that our conclusions only pertain to situations where the data exhibit the random-effects structure assumed by the LMM. A major critique against LMM is that the assumptions it makes regarding random effects may not be met in natural data, which could bias results (McNeish et al., 2017). While we tested an LMM with incorrect fixed effects (Figures 9, 10), and the nonlinear simulations violated assumptions of linearity of all methods, we never looked at the performance of a LMM with incorrectly specified random effects. Furthermore, one expectation of longitudinal data is that measurements are more correlated with temporally close measurements than with those taken at distant time points (Gibbons et al., 2010; Garcia and Marder, 2017). Our simulated data did not reflect this as the data points were equally correlated across various time points (and our LMM and GEE models, implemented with a random intercept and “exchangeable” working correlation structure, reflected the dependence structure of the simulated data). However, it would be of benefit to researchers to understand how incorrectly specifying the within-group correlation structure biases results. Both the LMM and GEE can be implemented with various within-group correlation structures, and GEE is reportedly robust to misspecifications of its working correlation matrix (Garcia and Marder, 2017). The simulated data also did not reflect the attrition that occurs in longitudinal studies, which would result in unbalanced repeated measures. Future simulation studies could focus on simulating more “realistic” longitudinal data and examine how the misspecification of LMM and GEE models affects analysis.

A limitation of the application to our natural cohort is that the GWAS summary statistics used to compute the PRS in African-ancestry participants had a smaller sample size (∼15,000) than that used for the European-ancestry PRS (∼175,000). The African-ancestry PRS is underpowered relative to the European-ancestry PRS, so the lack of significant findings for the PTSD-PRS in any method applied to African-ancestry participants may be due to this technical limitation. We also reiterate that the results found in our cohort, relating the PRS, PTE, CSA, and income to PTSD symptoms, are intended to showcase the variability of results among longitudinal data analysis methods applied to non-simulated data. A more thorough investigation of the genetic and environmental risk factors associated with PTSD development would need to consider (1) more psychosocial confounders, (2) competing models of best fit with regards to time-varying effects, and (3) alternative summary statistics for the African-ancestry PRS. However, our findings do suggest that a failure to replicate results could be due to the fact that various approaches, despite being adequate methods for the data under study, produce minute differences in findings. Furthermore, researchers should not fit multiple methods to their data and then choose the one with the most significant results or which validates their hypotheses.

By demonstrating model behavior under different simulated scenarios, we showed where serious issues such as FPR inflation or inaccurate estimations are likely to occur. If researchers observe features in their data that cause such drawbacks, they can choose a method that alleviates them. Our simulations evaluated method performance in multiple situations, including the analysis of a time-invariant predictor, which is standard for genetics. Therefore, these results can be directly applied to studies investigating the longitudinal effects of PRSs. We also showed discrepancies between results in natural data to highlight the practical impacts of the method choice on result interpretation. Longitudinal analyses are an important tool for genetic epidemiology as they provide methods to investigate how genetics play a role in the development or prognosis of diseases and disorders. However, to take advantage of these methods requires a clear understanding of the available methodology. Our findings can be utilized to develop experimental designs and select the optimum model with regard to accuracy, precision, power, and FPR. With this article, we provide a tool to researchers to further the goal of determining the genetic and nongenetic underpinnings of how complex diseases develop. Applying the appropriate LDA approach will foster reliable analyses that identify the risk factors contributing to the progression of diseases and disorders.

## Data Availability

Original datasets simulated in this study are publicly available at https://github.com/HallLab/ldasimulations. Restrictions apply to the existing natural longitudinal cohort dataset used in this article. It is restricted from public access as it contains sensitive information that could compromise research participant privacy and confidentiality. Individual-level data cannot be provided due to the confidentiality agreement with participants. Requests for access should be directed to jennie_noll@urmc.rochester.edu.
