# Supplementary material for: Longitudinal method comparison: modeling polygenic risk for post-traumatic stress disorder over time in individuals of African and European ancestry
Source: Front Genet. 2024 May 16;15:1203577. doi: 10.3389/fgene.2024.1203577 (PMC11137250; doi:10.3389/fgene.2024.1203577)

Supplementary Material

Longitudinal method comparison: Modeling polygenic risk for posttraumatic stress disorder over time in individuals of African and European ancestry

Kristin Passero, Jennie G. Noll, Shefali Setia Verma, Claire Selin, Molly A. Hall^*^

*** Correspondence:** Molly A. Hall: mah546@psu.edu

# Supplementary Figures and Tables

**Supplementary Figure 1. Prototypical simulated data utilizing a (A)** $\boldsymbol{Bin(n,0.5)}$ **or (B)** $\boldsymbol{N}\left( \boldsymbol{0,1} \right)$ **predictor**. Ten clusters with four waves were simulated. Time is plotted in one-unit increments along the x-axis; the response is plotted along the y-axis. The trajectory color defines the value of *X* (scales not shown). Panel columns reflect whether the predictor varied across time. Panel rows reflect the relationship between the predictor/time and the response. All β coefficients are 1. The response scales (y-axis) vary freely, due to the differing response magnitudes among linear, exponential, or parabolic simulations.


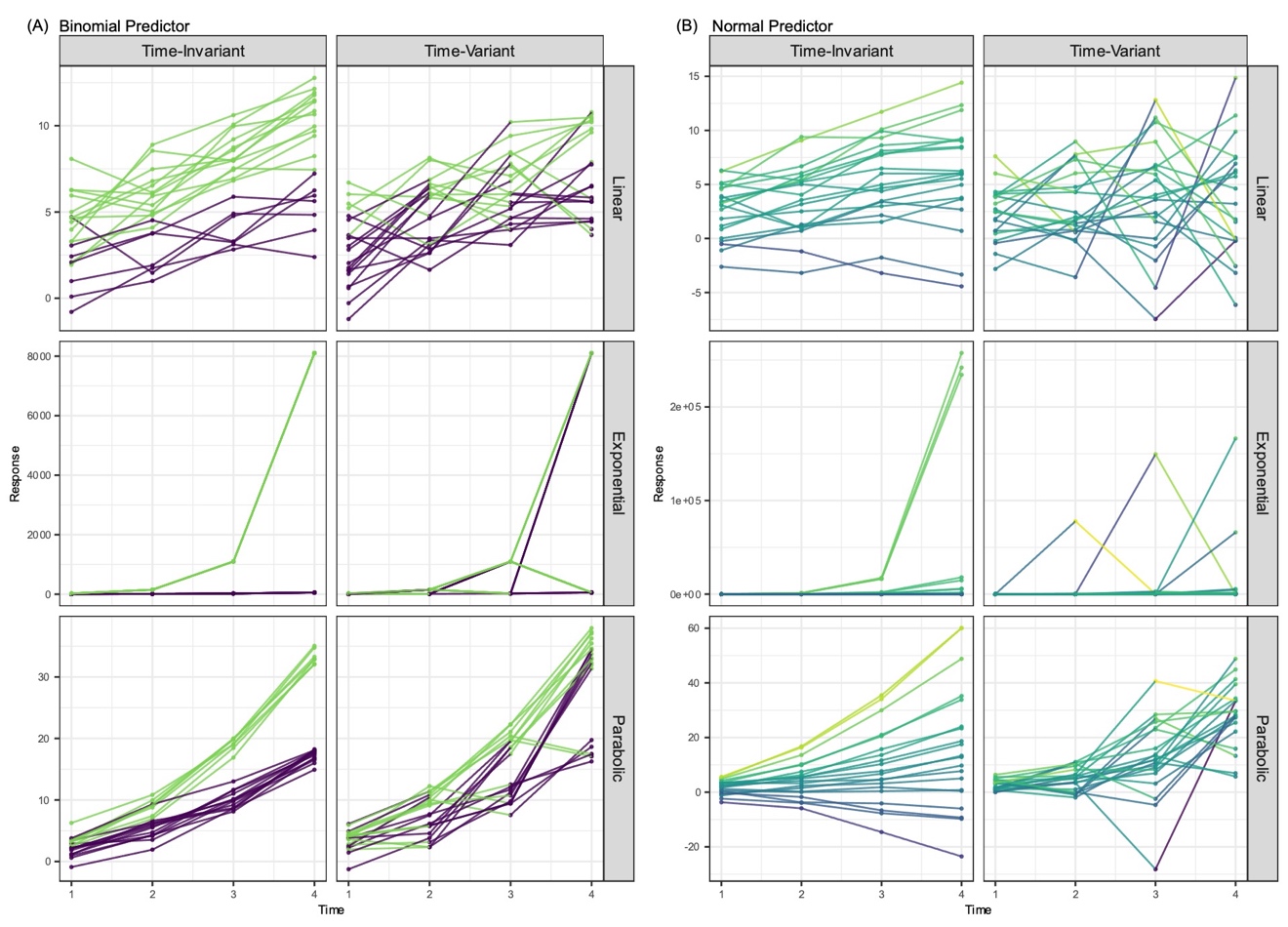


**Supplementary Figure 2.** **Estimation of** $\boldsymbol{Bin}\left( \boldsymbol{n,0.5} \right)$ **predictor in linear (A) and exponential (B) simulations.** The x-axis indicates median estimate difference. The y-axis indicates median absolute deviation (MAD); the y-axis range varies by panel. Point color and shape represent effect size (β) and the number of clusters, respectively. For the exponential simulation (B), the effect size of 0.3 is excluded. The dashed vertical and horizontal lines indicate a median or MAD of zero, respectively. Panel columns correspond to predictor time-variance and the intraclass correlation coefficient (ICC). Methods are plotted along panel rows. NLR=naïve linear regression, AGG=aggregate regression, FE=fixed effects model, LMM=linear mixed model, GEE=generalized estimating equation


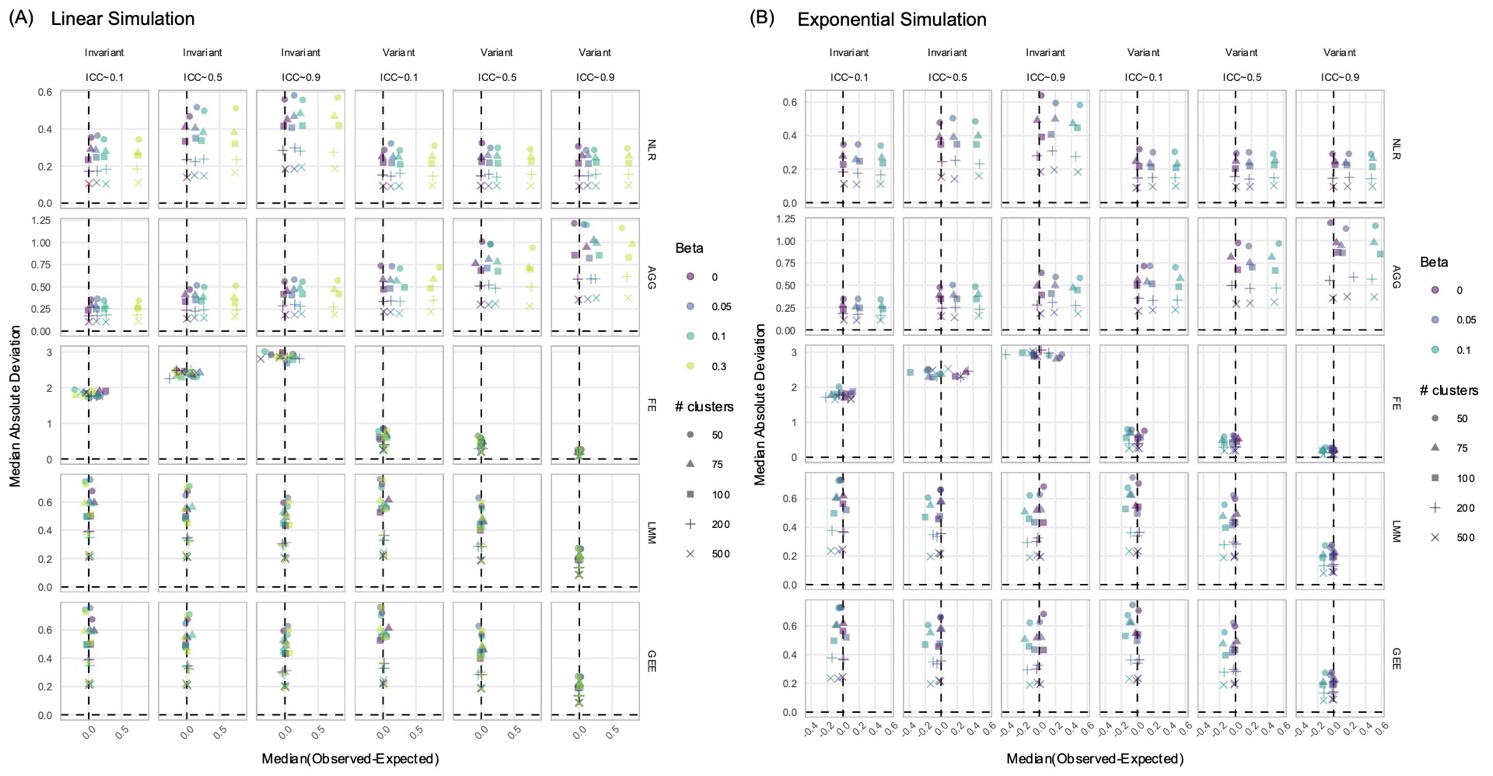


**Supplementary Figure 3.** **Estimation of (A)** $\boldsymbol{Bin(n,0.5)}$ **or (B)** $\boldsymbol{N}\left( \boldsymbol{0,1} \right)$ **predictor in exponential simulations at all effect sizes.** The x-axis indicates median estimate difference. The y-axis indicates median absolute deviation (MAD); the y-axis range varies by panel. Point color and shape represent effect size (β) and the number of clusters, respectively. The dashed vertical and horizontal lines indicate a median or MAD of zero, respectively. Panel columns correspond to predictor time-variance and the intraclass correlation coefficient (ICC). Methods are plotted along panel rows. NLR=naïve linear regression, AGG=aggregate regression, FE=fixed effects model, LMM=linear mixed model, GEE=generalized estimating equation


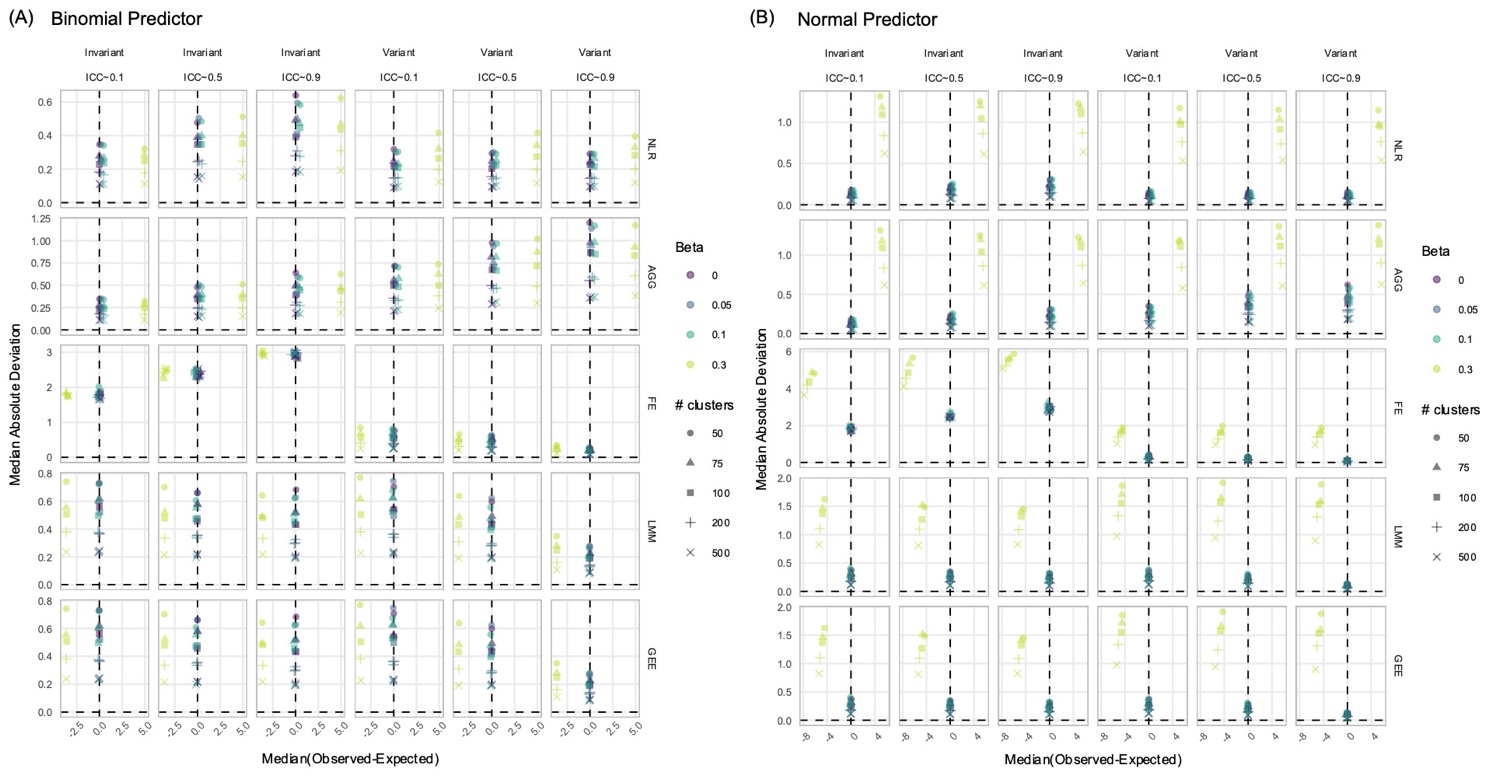


**Supplementary Figure 4.** **Estimation of** $\boldsymbol{Time}$ **in (A) linear, (B) exponential, and (C) parabolic simulations with a** $\boldsymbol{Bin(n,0.5)}$ **predictor.** The x-axis indicates median estimate difference. The y-axis indicates median absolute deviation (MAD); the y-axis range varies by panel. Point color and shape represent effect size (β) and the number of clusters, respectively. For the exponential simulation (B), the effect size of 0.3 is excluded. The dashed vertical and horizontal lines indicate a median or MAD of zero, respectively. Panel columns correspond to predictor time-variance and the intraclass correlation coefficient (ICC). Methods are plotted along panel rows. FE=fixed effects model, LMM=linear mixed model, GEE=generalized estimating equation


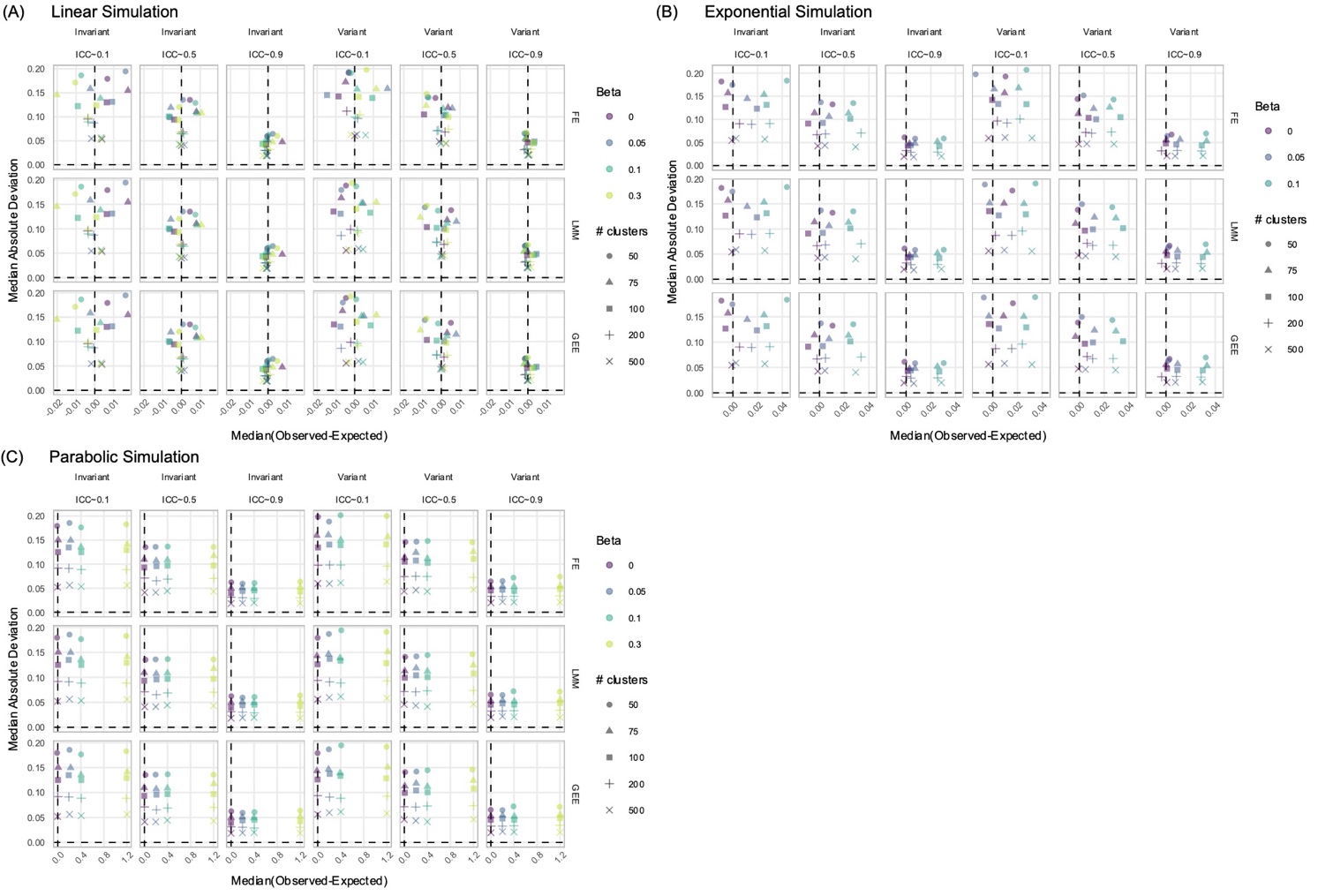


**Supplementary Figure 5.** **Estimation of** $\boldsymbol{Time}$ **with a (A)** $\boldsymbol{Bin}\left( \boldsymbol{n,0.5} \right)$ **or (B)** $\boldsymbol{N}\left( \boldsymbol{0,1} \right)$ **predictor in exponential simulations at all effect sizes.** The x-axis indicates median estimate difference. The y-axis indicates median absolute deviation (MAD); the y-axis range varies by panel. Point color and shape represent effect size (β) and the number of clusters, respectively. The dashed vertical and horizontal lines indicate a median or MAD of zero, respectively. Panel columns correspond to predictor time-variance and the intraclass correlation coefficient (ICC). Methods are plotted along panel rows. NLR=naïve linear regression, AGG=aggregate regression, FE=fixed effects model, LMM=linear mixed model, GEE=generalized estimating equation


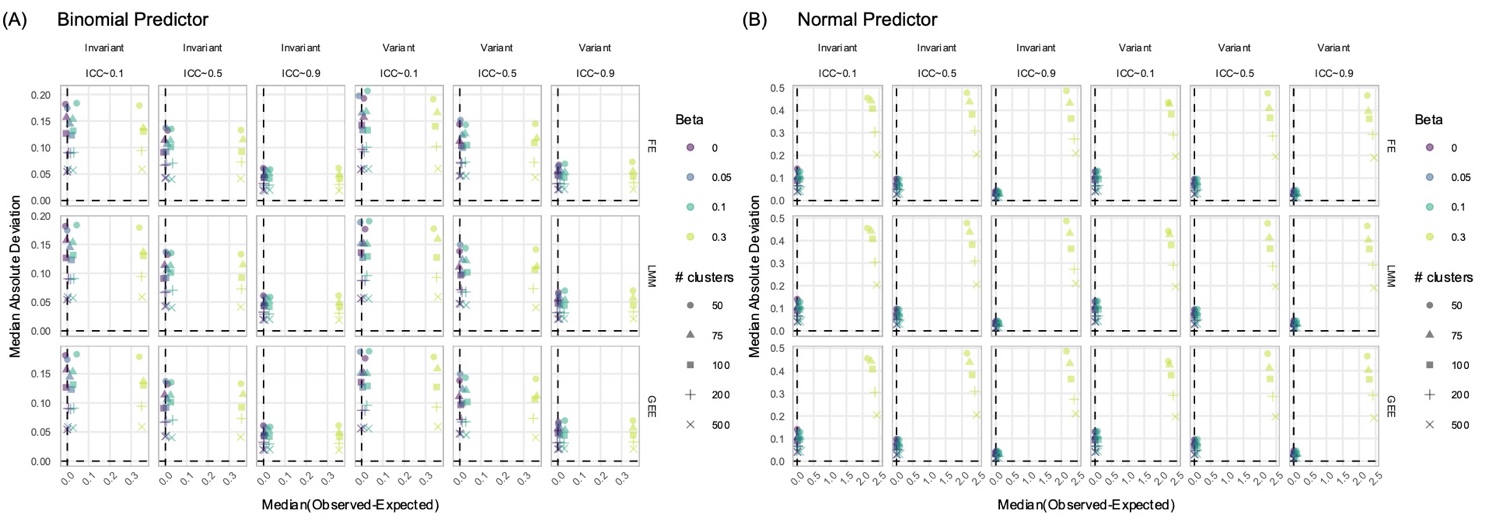


**Supplementary Figure 6.** **Estimation of** $\boldsymbol{X\times Time}$ **in (A) linear, (B) exponential, and (C) parabolic simulations with an** $\boldsymbol{N}\left( \boldsymbol{0,1} \right)$ **predictor.** The x-axis indicates median estimate difference. The y-axis indicates median absolute deviation (MAD); the y-axis range varies by panel. Point color and shape represent effect size (β) and the number of clusters, respectively. For the exponential simulation (B), the effect size of 0.3 is excluded. The dashed vertical and horizontal lines indicate a median or MAD of zero, respectively. Panel columns correspond to predictor time-variance and the intraclass correlation coefficient (ICC). Methods are plotted along panel rows. FE=fixed effects model, LMM=linear mixed model, GEE=generalized estimating equation


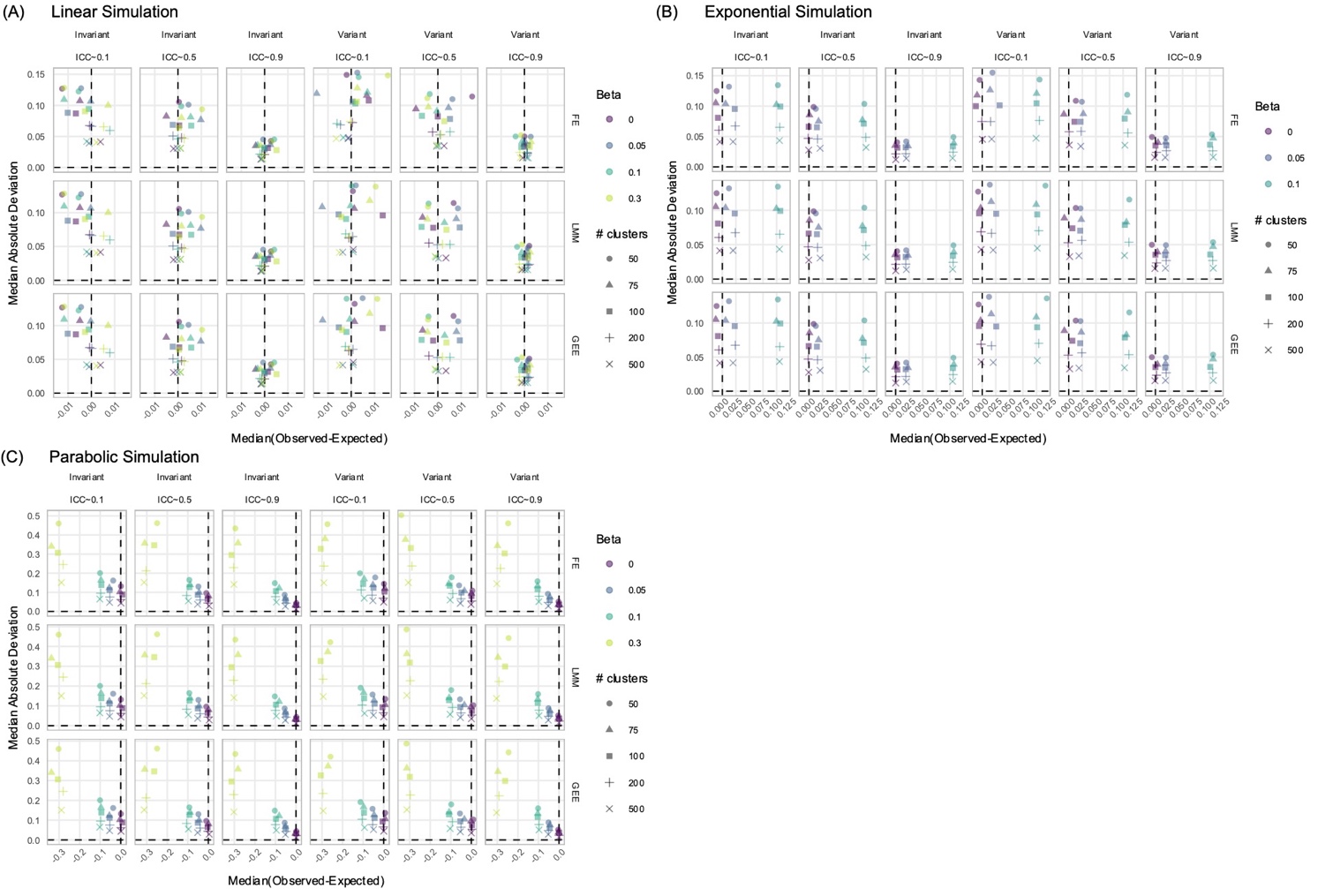


**Supplementary Figure 7.** **Estimation of** $\boldsymbol{X\times Time}$ **in (A) linear, (B) exponential, and (C) parabolic simulations with a** $\boldsymbol{Bin}\left( \boldsymbol{n,0.5} \right)$ **predictor.** The x-axis indicates median estimate difference. The y-axis indicates median absolute deviation (MAD); the y-axis range varies by panel. Point color and shape represent effect size (β) and the number of clusters, respectively. For the exponential simulation (B), the effect size of 0.3 is excluded. The dashed vertical and horizontal lines indicate a median or MAD of zero, respectively. Panel columns correspond to predictor time-variance and the intraclass correlation coefficient (ICC). Methods are plotted along panel rows. FE=fixed effects model, LMM=linear mixed model, GEE=generalized estimating equation


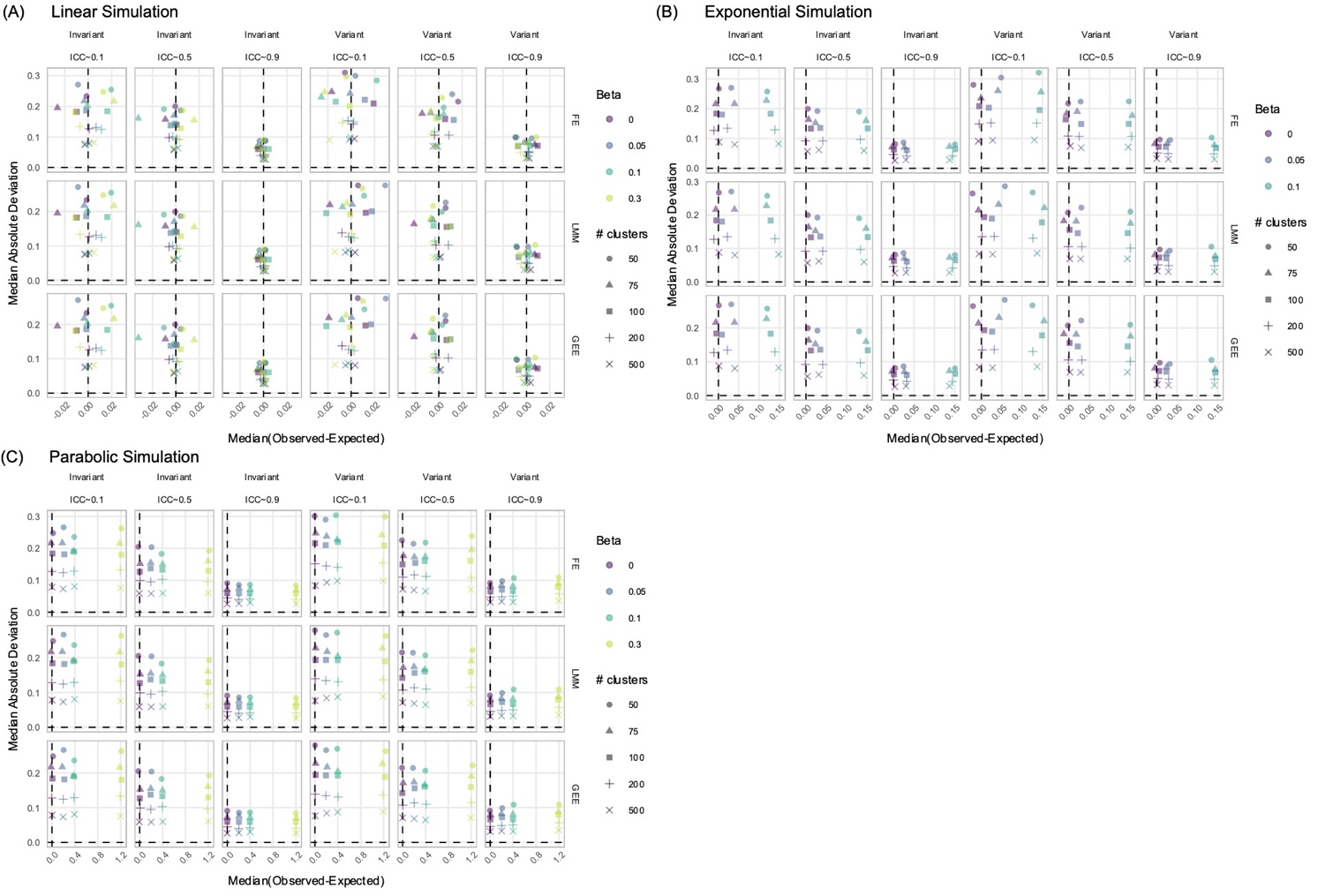


**Supplementary Figure 8.** **Estimation of** $\boldsymbol{X\times Time}$ **with a (A)** $\boldsymbol{Bin}\left( \boldsymbol{n,0.5} \right)$ **or (B)** $\boldsymbol{N}\left( \boldsymbol{0,1} \right)$ **predictor in exponential simulations at all effect sizes.** The x-axis indicates median estimate difference. The y-axis indicates median absolute deviation (MAD); the y-axis range varies by panel. Point color and shape represent effect size (β) and the number of clusters, respectively. The dashed vertical and horizontal lines indicate a median or MAD of zero, respectively. Panel columns correspond to predictor time-variance and the intraclass correlation coefficient (ICC). Methods are plotted along panel rows. FE=fixed effects model, LMM=linear mixed model, GEE=generalized estimating equation


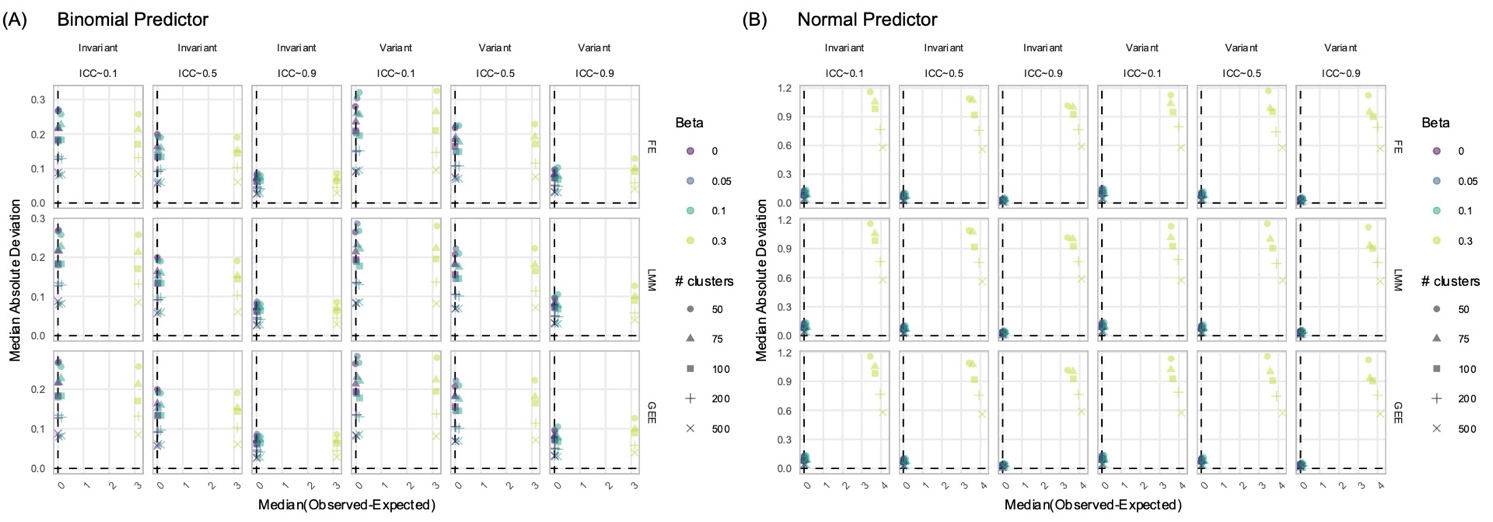


**Supplementary Figure 9. False positive rate (FPR) in simulations with an** $\boldsymbol{N}\left( \boldsymbol{0,1} \right)$ **predictor.** The x-axis indicates the number of clusters. The y-axis indicates the FPR. The shaded grey region is Bradley’s liberal FPR region from 2.5% – 7.5%. The panel columns correspond to the simulation’s linearity. The panel rows correspond to the intraclass correlation coefficient (ICC) and the predictor’s time-variance. Each method has an FPR trajectory, color-coded according to the legend. The line type denotes the variable. NLR=naïve linear regression, CRSE=cluster-robust standard error, AGG=aggregate regression, FE=fixed effects model, LMM=linear mixed model, GEE=generalized estimating equation


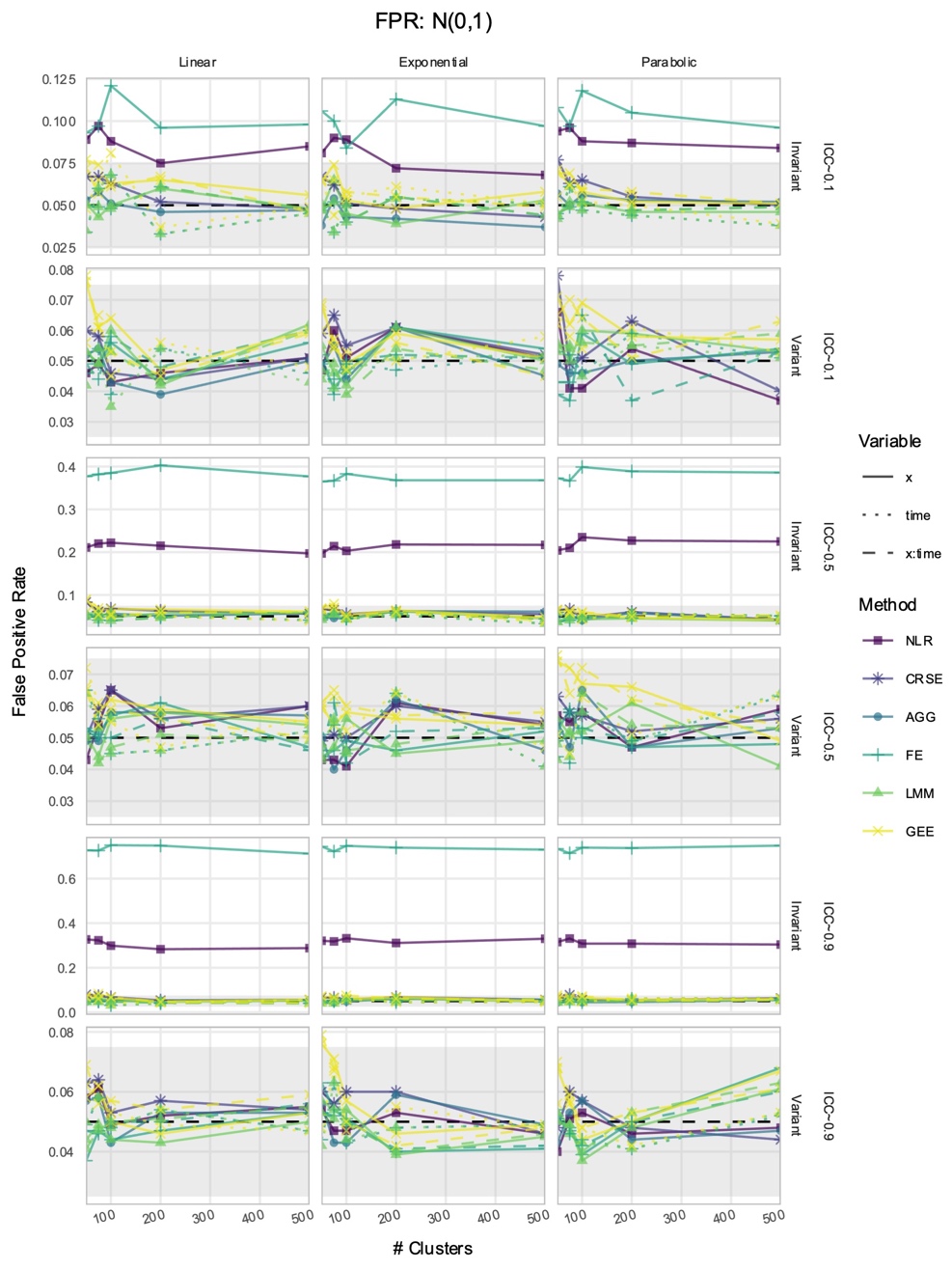


**Supplementary Figure 10. Estimation in exponential simulations with a correctly specified CRSE model at all effect sizes.** The x-axis indicates median estimate difference. The y-axis indicates median absolute deviation (MAD); the y-axis range varies by panel. Point color and shape represent effect size (β) and the number of clusters, respectively. The dashed vertical and horizontal lines indicate a median or MAD of zero, respectively. Panel columns denote the simulation linearity and intraclass correlation coefficient (ICC). Panel rows correspond to the variable and method. NLR=naïve linear regression, CRSE=cluster-robust standard error, LMM= linear mixed model


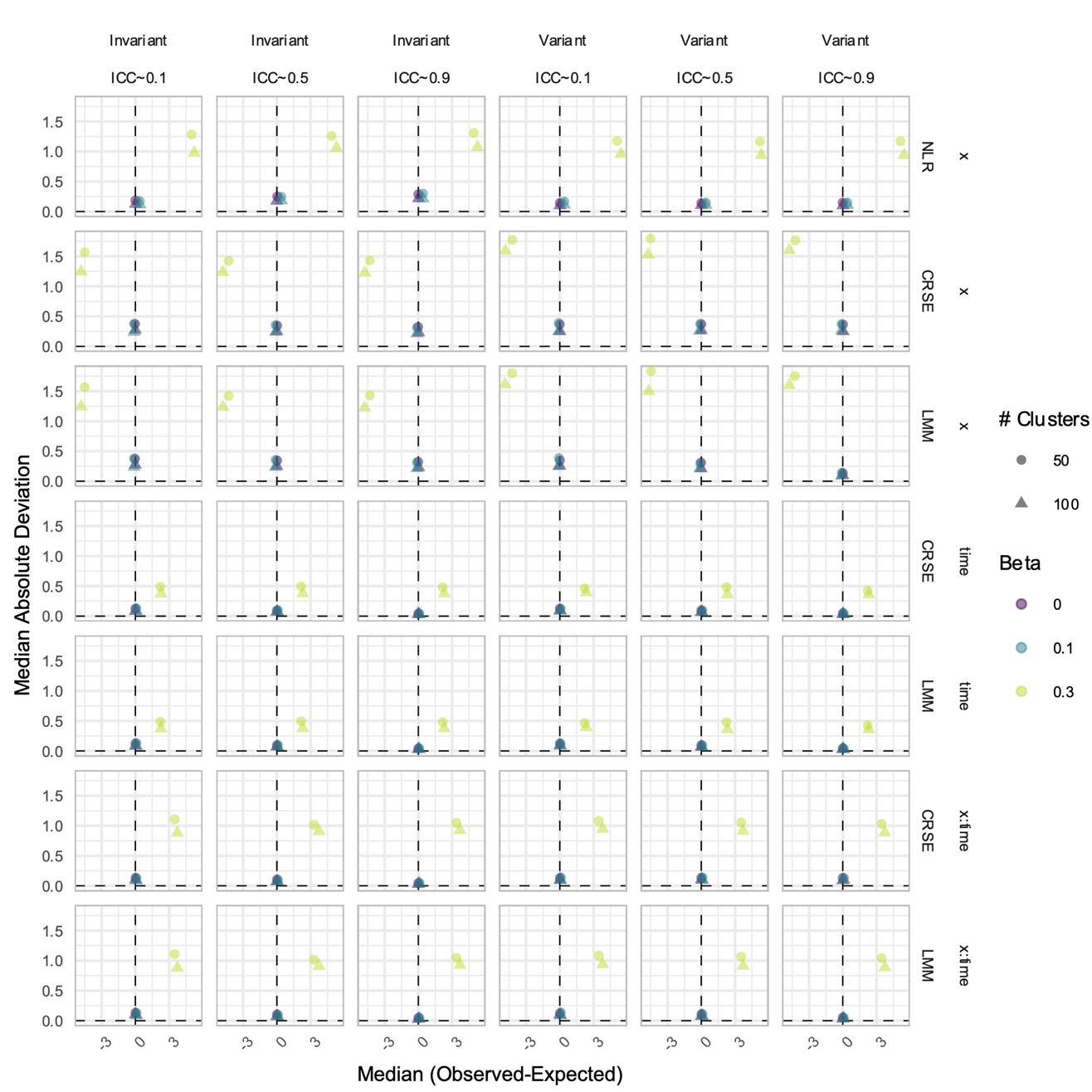

Supplement: Supplementary file 1 [file Table1.DOCX]
